# Viral Infections Exacerbate FUS-ALS Phenotypes in iPSC-Derived Spinal Neurons in a Virus Species-Specific Manner

**DOI:** 10.3389/fncel.2019.00480

**Published:** 2019-10-22

**Authors:** Jessica Bellmann, Anne Monette, Vadreenath Tripathy, Anna Sójka, Masin Abo-Rady, Antje Janosh, Rajat Bhatnagar, Marc Bickle, Andrew J. Mouland, Jared Sterneckert

**Affiliations:** ^1^Center for Regenerative Therapies Dresden, Technische Universität Dresden, Dresden, Germany; ^2^Lady Davis Institute for Medical Research, Jewish General Hospital, Montreal, QC, Canada; ^3^Department of Medicine, McGill University, Montreal, QC, Canada; ^4^Max Planck Institute of Molecular Cell Biology and Genetics, Dresden, Germany; ^5^Verge Genomics, San Francisco, CA, United States

**Keywords:** amyotrophic lateral sclerosis, induced pluripotent stem cells, FUS, rabies virus, HIV-1, Zika virus

## Abstract

Amyotrophic lateral sclerosis (ALS) arises from an interplay of genetic mutations and environmental factors. ssRNA viruses are possible ALS risk factors, but testing their interaction with mutations such as in *FUS*, which encodes an RNA-binding protein, has been difficult due to the lack of a human disease model. Here, we use isogenic induced pluripotent stem cell (iPSC)-derived spinal neurons (SNs) to investigate the interaction between ssRNA viruses and mutant *FUS*. We find that rabies virus (RABV) spreads ALS phenotypes, including the formation of stress granules (SGs) with aberrant composition due to increased levels of FUS protein, as well as neurodegeneration and reduced restriction activity by FUS mutations. Consistent with this, iPSC-derived SNs harboring mutant *FUS* are more sensitive to human immunodeficiency virus (HIV-1) and Zika viruses (ZIKV). We demonstrate that RABV and HIV-1 exacerbate cytoplasmic mislocalization of FUS. Our results demonstrate that viral infections worsen ALS pathology in SNs with genetic risk factors, suggesting a novel role for viruses in modulating patient phenotypes.

## Introduction

Amyotrophic lateral sclerosis (ALS), a progressive neurodegenerative disease specifically affecting motor neurons (MNs), has an average age of onset of about 55 years (Talbott et al., 2016). Patients typically suffer increasing paralysis and mortality about 3–5 years after diagnosis. The only FDA-approved drugs are riluzole, which inhibits excitotoxicity and edaravone, which is an antioxidant (Jaiswal, 2019). However, both of these therapeutics have relatively minor effects on survival (Jaiswal, 2019), and more effective treatments are urgently needed. Studies of twin siblings have demonstrated that ALS is caused by a combination of genetic and environmental factors, and the environmental component comprises about 40% of the risk of developing the disease (Graham et al., 1997; Al-Chalabi et al., 2010). About 10% of ALS cases are familial (fALS), and the identification of causative mutations has provided critical insight into the genetic component of ALS. At present, mutations in a large number of proteins have been associated with ALS (Cook and Petrucelli, 2019). A striking number of these, including fused in sarcoma (FUS), are RNA binding proteins (RBPs) that contain prion-domains, are recruited to stress granules (SGs), and are prone to protein aggregation when mutated (Johnson et al., 2009; Liu-Yesucevitz et al., 2010; Couthouis et al., 2011, 2012; Kim et al., 2013; Li et al., 2013). ALS-associated mutations in all of these proteins leads to their increased cytoplasmic accumulation and recruitment to SGs. This aberrant composition of SGs causes a liquid-to-solid phase transition, which is believed to nucleate protein aggregates that are pathological hallmarks of ALS. Thus, SGs have been called crucibles of ALS (Li et al., 2013). Abnormal aggregates of RBPs tend to spread along neuronal circuitry in a corticofugal manner (Braak et al., 2013, 2017). Since small amounts of aggregated RBPs such as TDP-43 and FUS also induce aggregation of soluble wild type proteins, it has been suggested that ALS-associated RBP aggregates behave like prions (Furukawa et al., 2011; Polymenidou and Cleveland, 2011; Nomura et al., 2014). Although most studies have focused on fALS, RBP aggregation is a pathological hallmark of sporadic ALS (Li et al., 2013), suggesting that RBPs play critical roles in sporadic ALS as well as in familial cases.

Multiple lines of evidence implicate viruses as environmental risk factors for ALS. Initially, it was found that reverse transcriptase activity was increased in ALS patients compared with healthy individuals, suggesting that infection by retroviruses might be risk factors for ALS (Viola et al., 1975; Andrews et al., 2000; Steele et al., 2005; MacGowan et al., 2007; McCormick et al., 2008). Subsequently, it was observed that cases of chronic poliomyelitis resemble ALS, suggesting that chronic enterovirus (EV) infection could be linked to ALS pathology (Norris, 1977; Ravits, 2005). Several cases of ALS-like phenotypes have been reported in individuals infected with human immunodeficiency virus (HIV-1), where motor symptoms were observed to be ameliorated by anti-viral drugs (Alfahad and Nath, 2013). Some studies have reported increased EV levels in ALS patients compared with controls (Woodall et al., 1994; Berger et al., 2000; Giraud et al., 2001; Vandenberghe et al., 2010). One study found that human herpes virus (HHV) increased the risk of ALS (Cermelli et al., 2003). Thus, viral infections likely represent an environmental risk factor of ALS pathogenesis.

We hypothesize that viral infections exacerbate the pathogenesis of inherited genetic risk factors. This is supported by observations that similar cellular pathways are commonly shared between ALS and viral pathogenesis, including mitochondrial dysfunction (Manfredi and Xu, 2005; Khan et al., 2015), oxidative stress (Simpson et al., 2003; Limongi and Baldelli, 2016), ER stress (He, 2006; Hetz and Saxena, 2017), and SG formation (White and Lloyd, 2012; Droppelmann et al., 2014). In addition, numerous ALS-associated proteins have been shown to either directly interact with viral nucleic acids or to regulate the cellular response to viral infection, including FUS, hnRNPA1, hnRNPA2, SETX, TBK1, and TDP-43 (Ou et al., 1995; Sharma et al., 2003; Beriault et al., 2004; Miller et al., 2015; Dunker et al., 2018). In addition, SGs, which have been suggested to be crucibles of establishment of ALS pathogenesis, play an essential role in the anti-viral defense of cells (Lloyd, 2012). Therefore, understanding the molecular mechanisms of this co-pathogenic interaction may lead to novel therapeutic approaches against ALS pathogenesis.

In this manuscript, we use induced pluripotent stem cell (iPSC)-derived cultures of spinal neurons (SNs), which are enriched in MNs, to demonstrate that RNA viruses exacerbate ALS phenotypes in neurons harboring the ALS-associated mutation P525L *FUS*. Neuronal connectivity is essential for function, and it has been shown that maintenance of MN connectivity is essential for preventing ALS phenotypes (Parone et al., 2013). Therefore, we used monosynaptic rabies tracing to assess neuronal connectivity of iPSC-derived neurons with either wild type (WT) or P525L FUS-eGFP. However, we unexpectedly found that RABV spreads ALS phenotypes between neurons, including the formation of aberrant SGs as well as neurodegeneration, and suggesting that viral spreading exacerbates the spreading of ALS pathogenesis. Interestingly, in addition to being an ALS-associated RBP, FUS negatively regulates Kaposi’s sarcoma-associated herpesvirus gene expression (Dunker et al., 2018), demonstrating that FUS is a viral restriction factor. Similarly to retroviruses and EV, which have been linked to ALS pathogenesis, RABV also has an ssRNA genome. RABV induces SG formation, which have been linked to the formation of pathological aggregates in ALS patients (Nikolic et al., 2016). Consistent with the notion that FUS may act as a RABV restriction factor, we observed that iPSC-derived SNs with mutant *FUS* showed significantly higher expression of the RABV-mCherry transgene, as well as increased neurodegeneration following RABV infection compared with isogenic controls. Since RABV has not been linked to ALS pathogenesis, we tested additional viruses. We show that iPSC-derived SNs harboring mutant *FUS* are also more sensitive to HIV-1 and Zika viruses (ZIKV). Finally, we demonstrate that RABV and HIV-1 induce mislocalization of FUS, exacerbating the effects of the P525L mutation. Our results demonstrate that viral infections exacerbate ALS pathology in SNs predisposed with genetic ALS risk factors, suggesting a novel role for viruses in modulating patient phenotypes, and perhaps even being responsible for onset of aggressive ALS pathology in patients having inherited ALS risk.

## Materials and Methods

### Ethics Statement

All procedures involving human participants were performed in accordance with the ethical standards of the institutional and/or national research committee as well as with the 1964 Helsinki declaration and its later amendments.

### Cell Culture

Induced pluripotent stem cells used in this project were previously generated and characterized (Marrone et al., 2018). For the derivation of spinal neuron precursor cells, iPSCs were cultured on a 6-well plate until they reached 80% confluency. The cells were incubated with collagenase IV (Thermo Fisher) at 37°C. Pieces of colonies were collected by sedimentation and resuspended in human ESC medium [KnockOut-DMEM (Thermo Fisher) supplemented with 20% serum replacement (Thermo Fisher), 100 μg/ml penicillin-streptomycin (Biochrom) and 2 mM L-glutamine (Biochrom) (PSG), 1% non-essential amino acids (Merck), and 0.1 mM β-mercaptoethanol (Gibco)] supplemented with 200 μM ascorbic acid (AA, Sigma), 3 μM CHIR99021 (CHIR, Axon), 0.5 μM dorsomorphin (Selleckchem), 10 μM SB-431542 (SB, Biomol), and 5 μM Y-27632 dihydrochloride (Y, Abcam) and plated in a 10 cm petri dish (Sarstedt). After 4 days, medium was replaced with DMEM/F-12 and Neurobasal medium (1:1) supplemented with PSG, N2, and B27 supplement (N2B27, Thermo Fisher) and additionally supplemented with 200 μM AA, 3 μM CHIR, 0.5 μM purmorphamine (PMA, Cayman), 0.1 μM retinoic acid (RA, Sigma), 2 μM SB, 2 μM DMH-1 (Tocris) and maintained for another 2 days. The EBs formed in the suspension culture were disaggregated and plated on Matrigel (Corning) coated 12-well dishes. After 4–6 days, medium was replaced by SNP medium which contained 0.5 mM valproic acid (VPA, Cayman) in addition to the previous supplemental factors. Spinal neuron precursor cells were plated at a density of 3 × 10^5^ cells on a 12-well plate and cultured in N2B27 supplemented with 200 μM AA, 1 μM RA, 2 μg/ml glia-derived growth factor (GD, PeproTech), 1 μg/ml brain-derived growth factor (BD, PeproTech), and 0.5 μM smoothened agonist (Biomol). After 6 days, medium was exchanged to maturation medium consisting of N2B27 supplemented with 200 μM AA, 0.1 mM dibutyryl cyclic-AMP sodium salt (Sigma), 1.5 μg/ml transforming growth factor beta 3 (Peprotech), 4 μg/ml GD, 2 μg/ml BD, and 5 μg/ml activin A (aA, eBioscience). After 48 h, pre-differentiated SNs were replated inside the microfluidic chamber (Xona, RD900 with 900 μm long microgrooves) (6 × 10^4^ cells). SNs were matured by exchanging maturation medium, which lacked AA and with 0.1 μM γ-secretase inhibitor (compound E, Merck), every second day.

### Spinal Neuron Characterization

Expression levels of marker genes were determined by mRNA-sequencing (Amaryllis Nucleics, Oakland CA, United States). Reads were quantified at the transcript level using the software package Salmon (Patro et al., 2017) and the reference genome GRCH38^1^ and summarized at the gene level with the R package tximport (Soneson et al., 2015). Reported values are regularized log counts (Love et al., 2014). Cell types were classified by a support vector machine trained on reference cell types derived from mouse (Sun et al., 2015) with PCA-computed eigengenes as features.

### RABV, HIV-1, and ZIKV

Work with RABVΔG virus was performed according to an approved project protocol (2184: Project 32-02 und 2178: Project 52-6.1) in Biosafety Level 2 laboratories approved (54-845218412 and 54-845217817) by the Sächsisches Staatsministerium für Wissenschaft und Kunst (SMWK). All personnel (JB) were trained extensively in the handling and safety precautions when handling and disposing of infectious material. RABVΔG-mCherry was produced according to Osakada and Callaway (2013) using the pSADdeltaG-mCherry vector (Addgene, 32636). Packaging cell lines were provided by John Naughton (Salk Institute, United States). To generate RABVΔG without EnvA pseudotyping, the published protocol was discontinued after step 46, and the virus was concentrated via ultracentrifugation at 32.500 rpm for 3 h 30 min. To quantify the percentage of RABVΔG-mCherry positive cells, neurons were infected with RABVΔG at a multiplicity of infection (MOI) of 0.2 and analyzed using flow cytometry against FUS-eGFP and RABVΔG-mCherry double positive cells at 7 days following infection. To quantify stress granule formation, 2 days following infection with RABVΔG at a MOI of 0.5, neurons were immunostained and imaged using confocal microscopy for FUS-eGFP and TIAR granules. Cells in at least three frames were counted for each condition.

Work with HIV-1 and Zika virus was performed under strict Public Health Agency of Canada guidelines under the Pathogen and Toxin Licenses L-R3-01013-19 (Risk Group 3 Pathogens) and L-R2-14345-16 (Risk Group 2 Pathogens), respectively. All personnel (AM and AJM) were trained extensively in the handling and safety precautions when handling and disposing of infectious material. ZIKV stocks of the Canadian imported Thai ZIKV strain PLCal_ZV (GenBank accession KF993678.1), were prepared by passaging in Vero cells as previously described (Fonseca et al., 2014; Amorim et al., 2017; Pardy et al., 2017). Briefly, 6.0 × 10^6^ Vero cells were plated in a T182.5 flask, and were infected at an MOI of 0.5 in 10 ml of Eagle’s minimal essential medium (EMEM) (Wisent) for incubation (37°C, 5% CO_2_) for 2 h. Infection medium was replaced with ZIKV infection medium [DMEM supplemented with 2% FBS, 1% non-essential amino acids, 1% L-glutamine, 50 U/ml Penicillin and 50 μg/ml Streptomycin (Wisent), and 15 mM HEPES buffer (Sigma-Aldrich)]. After 2 days of infection, the cell supernatant was filtered with a 0.45 μm membraned filter, and viral stocks were titered using a plaque forming unit (PFU) assay. For the PFU assay, 6.0 × 10^5^ Vero cells were seeded in six-well plates and incubated (37°C, 5% CO_2_) overnight, prior to being infected by eight 1/10 serial dilutions of previously filtered viral supernatants diluted in EMEM (37°C, 5% CO_2_) for 2 h, after which, virus dilutions were replaced by CMC medium composed of EMEM with 1.2% carboxymethylcellulose (CMC) (Sigma-Aldrich), 2% FBS, 50 U/mL Penicillin, and 50 μg/ml Streptomycin (37°C, 5% CO_2_) for 4 days. Cells were washed with PBS, and were fixed using 4% paraformaldehyde (PFA) in PBS for 20 min, were washed with ddH_2_O, and incubated at room temperature (RT) with a 0.1% crystal violet solution for 30 min for plaque visualization. Viral titers were then calculated as number of plaques divided by the dilution factor times the infection volume.

Vesicular stomatitis virus G (VSV-G) pseudotyped HIV-1 was generated exactly as described (Mouland et al., 2002) and used to infect iPSC-derived SNs at the indicated MOI.

### Monosynaptic Tracing Using RABVΔG (EnvA)

The pBOB-synP-HTB plasmid, a lentiviral vector for the generation of the starter neurons for monosynaptic rabies tracing, was purchased from Addgene (number 30195). For the infrared fluorescent protein (iRFP) starter neurons, the GFP coding sequence in pBOB-synP-HTB was replaced by iRFP (provided by the Mansfeld group, BIOTEC, Germany). For lentiviral production, pBOB-synP-HTB (either GFP or iRFP) was separately transfected into HEK293T cells together with VSV-G and Gag/Pol expression vectors (provided by the Caligari group, CRTD, Germany) using polyethylenimine. Supernatant was harvested after 48 and 96 h and concentrated by ultracentrifugation at 32,500 rpm for 3 h 30 min and resuspended in PBS. Starter neurons were infected and sorted against GFP or iRFP using the BD FACSAria III.

EnvA pseudotyped RABVΔG-mCherry [RABVΔG (EnvA)] was produced as described (Osakada and Callaway, 2013) using the pSADdeltaG-mCherry vector (Addgene, 32636). RABVΔG (EnvA) was concentrated via ultracentrifugation at 32.500 rpm for 3 h 30 min. Titering was performed using TVA-expressing HEK293T cells (provided by John Naughton at the Salk Institute, United States) as described in the protocol. Titers were 2–3 × 10^7^ TU/ml. For tracing experiments using microfluidic chambers (Xona, RD900 with 900 μm long microgrooves) 2 × 10^5^ GFP starter neurons were plated in one compartment and FUS-eGFP neurons in the opposed compartment at maturation day 3. After 2 or 4 weeks, starter neurons were infected with RABVΔG(EnvA) at a MOI of 0.02 to the respective compartment. After 1 week, neurons were fixed using 4% PFA. Chambers were washed twice with PBS and three times with 0.1% BSA in PBS with 0.005% Tween-20, including Hoechst counterstaining for nuclei in the second washing step. Tile scans with z-stacks were captured using the laser scanning confocal microscope (Zeiss LSM780/FCS). Images of the starter neuron compartment were analyzed for percentage of mCherry and GFP positive neurons and images of the traced neuron compartment for percentage of mCherry positive neurons using cell profiler and KNIME software. The percentage of traced cells were normalized to number of mCherry positive starter cells. For tracing experiments using flow cytometry, 2.5 × 10^5^ iRFP starter neurons were combined with 2.5 × 10^5^ FUS-eGFP neurons in one well of a Matrigel coated 24-well dish. The co-culture was infected with RABVΔG (EnvA) at a MOI of 0.02 and analyzed using flow cytometry. For that, co-cultures were treated with Accutase for 15–30 min and resuspended in PBS supplemented with 0.1% BSA, 2 mM EDTA and 5 μM Y-27632 dihydrochloride. Cells were analyzed using LSR Fortessa. Infected starter neurons were detected by first, gating the iRFP-positive cell population and second, counting the number of mCherry-positive cells. Gating was done using control neurons. The traced neurons were quantified by gating FUS-eGFP and RABVΔG-mCherry double-positive cells. The percentage of traced cells were normalized to number of mCherry positive starter cells.

### Lactate Dehydrogenase (LDH) Release Assay

Neurons were seeded at a density of 4 × 10^4^ neurons per well of a 96-well plate and cultured for at least 2 weeks. Triplicate wells of neurons were infected with RABVΔG at a MOI of 0.5. The supernatant was collected 48 h after infection and LDH release was measured using the LDH Cytotoxicity Detection Kit (Takara) according to the manufacturer’s instructions. The signal was quantified using a Synergy Neo plate reader (BioTek).

### Cell Viability Assay (MTT Assay)

3-[4,5-Dimethylthiaoly]-2,5-diphenyltetrazolium bromide (MTT) (Sigma Aldrich) assay was performed to detect proliferation of SNs that were uninfected or infected by HIV-1 or ZIKV. 96-well flat bottom plates were pre-coated with Corning^®^ Matrigel^®^ Matrix High Concentration (HC), Growth Factor Reduced (GFR) ^∗^LDEV-free treated according to the manufacturer’s recommendations. Differentiated iPSC-derived SNs were plated at 7 × 10^4^ per well of 96-well plates in 100 μL NS5B maturation medium, and were infected by HIV-1 or ZIKV in triplicate at MOIs of 0, 0.25, 0.5, or 1 for incubation (37°C, 5% CO_2_) for 2 h, prior to replacing with fresh NS5B maturation medium and incubating (37°C, 5% CO_2_) for another 24 h. The MTT assay was then carried out as recommended by the manufacturer, where media was replaced with freshly diluted MTT in PBS for a final concentration of 0.5 mg/mL, and plates were incubated (37°C, 5% CO_2_) for 4 h prior to addition of the Solubilization solution, Plates were incubated (37°C, 5% CO_2_), and absorbance was measured at 590 nm by a microplate reader (Bio-Rad).

### Immunofluorescence and FISH

Cells were fixed for 20 min at RT in 4% PFA in PBS. Permeabilization and blocking of non-specific epitopes was performed simultaneously using 0.1% Triton X-100, 1% BSA, and 10% FBS in PBS for 45 min. Subsequently, the primary antibodies [mouse anti tubulin beta 3 (TUBB3) (1:1000, BioLegend, BLD-801202), cleaved caspase 3 (CC3) (cell signaling, 9661s, 1:300), MAP2 (1:5000, Abcam, ab92434), TIAR (1:500, BD, 610352), SMI-32 (1:500, Millipore, NE1023), CHAT (1:400, Millipore, AB144P), ISLET-1 (1:1500, Abcam, ab20670)] were applied overnight at 4°C in 0.1% BSA in PBS. The next day, the cells were washed with 0.1% BSA in PBS and incubated with the secondary antibody for 1 h at RT. Finally, cells were washed three times with 0.1% BSA in PBS-T (0.005% Tween-20), including Hoechst counterstaining for nuclei in the second washing step. Cells were imaged either with a Zeiss ApoTome or a laser scanning confocal microscope (Zeiss LSM780/FCS).

Immunofluorescence and FISH analyses on glass cover slips have been described previously (Monette et al., 2011; Vyboh et al., 2012). Briefly, sterile glass coverslips in 12-well flat bottom plates were pre-coated with Corning Matrigel Matrix HC, GFR ^∗^LDEV-free. Differentiated iPSC-derived SNs were plated at 1 × 10^6^ per well in 500 μL NS5B maturation medium, and were infected by HIV-1 or ZIKV in triplicate at MOIs of 0 or 1 for incubation (37°C, 5% CO_2_) for 2 h, prior to replacing with fresh NS5B maturation medium and incubating (37°C, 5% CO_2_) for another 24 h. Cells were washed with PBS, and were fixed using 4% PFA in PBS for 20 min, then permeabilized with 0.2% Triton X-100 for 10 min, and washed twice and stored in PBS until immunostaining. For FISH analyses of the HIV-1 vRNA, coverslips were treated with DNase I (Invitrogen) for 30 min and washed. A digoxigenin-labeled RNA probe was synthesized via *in vitro* transcription from the plasmid pKS-Pol236nt in the presence of digoxigenin-labeled UTP from the RNA Labeling Mix (Roche) and was annealed to permeabilized cells overnight at 42°C. After hybridization, the vRNA was visualized by incubating cells on coverslips with a primary sheep anti-Digoxigenin antibody (1:200, Roche, Sigma-Aldrich, 11333089001) for 1 h at 37°C, washing with PBS, followed by incubation with a donkey anti-sheep IgG (H + L) cross-adsorbed, Alexa Fluor^®^ 594 for 1 h at 37°C (1:400, Invitrogen, Thermo Fisher Scientific, A-11016). For immunostaining of proteins, cells on coverslips were incubated with the following primary antibodies against antigens of interest for 1 h at 37°C: ZIKV vRNA, mouse monoclonal J2 recognizing double-stranded RNA (dsRNA) intermediates of viruses (1:200, Scicons, Hungary, J2), rabbit polyclonal G3BP1 (1:500, Imed Gallouzi, McGill university). Coverslips were then washed with PBS, and incubated with corresponding secondary antibodies for 1 h at 37°C: donkey anti-mouse IgG (H + L) highly cross-adsorbed, Alexa Fluor^®^ 647, and donkey anti-rabbit IgG (H + L) highly cross-adsorbed, Alexa Fluor^®^ 594 (1:400, Invitrogen, Thermo Fisher Scientific, A-21207). Coverslips were stained with DAPI (Sigma-Aldrich), dried, and mounted onto slides using a mounting medium (Immu-mount; Thermo Fisher Scientific). Images were acquired at RT using a microscope (DM16000B; Leica) equipped with a spinning disk confocal head (WaveFX; Quorum), a 63X (1.4 numerical aperture oil immersion) plan apochromat objective lens, and an EM charge-coupled device camera (ImageEM; Hamamatsu Photonics). Volocity imaging acquisition software (PerkinElmer) was used, in which Alexa Fluor 633, 594, and 488 antibody fluorochromes and DAPI were scanned using excitation wavelengths of 646, 561, 491, and 405 nm, and emission spectra were filtered with 665– 715-, 570– 620-, 500– 550-, and 435–485-nm bandpass filters, respectively and sequentially. Images were recorded at a thickness of 0.2 μm and were digitized at a resolution of 1,024 × 1,024 pixels. Volocity was used to export.tiff files then imported into Imaris software (Andor) for pseudocoloring and colocalization analysis using Mander’s coefficient. Representative images were imported into Illustrator (Adobe) for figure montage, and GraphPad Prism 8 was used for graph building and calculations of all reported statistics, where reported statistical significance is *p* < 0.05.

### FUS-eGFP Granule and Translocation Analysis

To quantify FUS-eGFP stress granule intensity of RABV infected SNs, images were processed using FIJI, by first subtracting the background (rolling ball radius: 5 pixels) of the TIAR channel and applying a threshold (otsu) were SGs are visualized (threshold was the same between FUS-P525L and FUS WT conditions). A particle analysis was performed (size 1000 pixels). A mask was created from the particles, put on the FUS-eGFP channel and mean intensity measured. To quantify FUS-eGFP mean intensity, cytoplasmic and nuclear areas were selected and measured using FIJI. To quantify the nuclear to cytoplasmic ratio, cytoplasmic and nuclear intensity was measured and ratio of each cell quantified. Total FUS-eGFP levels were quantified by averaging nuclear and cytoplasmic mean intensities. Images of RABV, ZIKV, and HIV-1 infections were analyzed and compared with uninfected conditions.

## Results

### Differentiation of Isogenic FUS-eGFP iPSCs Into Spinal Neurons Enriched for MNs

**FIGURE 1:**
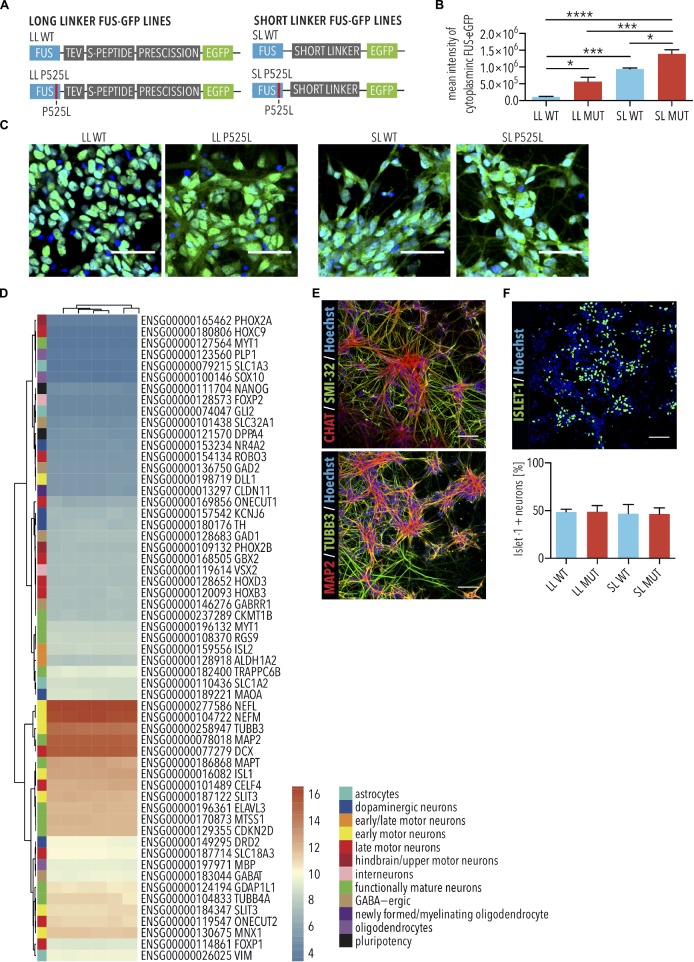
Characterization of FUS-eGFP induced pluripotent stem cell (iPSC)-derived spinal neurons. **(A)** Schematic view explaining long (LL) and short linker (SL) (gray) connecting eGFP (green) to the C-terminus of wild type and P525L FUS (blue). **(B,C)** Spinal neurons with LL and SL P525L FUS-eGFP lines show increased mislocalization of FUS-eGFP to the cytoplasm compared with WT controls and, previously shown (Marrone et al., 2019), SL enhances mislocalization by disrupting the function of the adjacent nuclear localization signal. Quantified by mean intensity of cytoplasmic GFP. Scale bar = 50 μm. *n* = 4. Error bars indicate SEM. ^∗^, ^∗∗∗^, and ^∗∗∗∗^ Correspond to *p* < 0.05, 0.001, and 0.0001, respectively, according to 1way ANOVA, Tukey post-test for multiple comparisons. **(D)** Transcriptome analysis shows that iPSC-derived spinal neurons express markers of multiple neuronal subtypes. **(E)** Immunostaining of iPSC-derived spinal neurons for neuronal markers (TUBB3 and MAP2) and motor neuron markers (CHAT and SMI-32). Scale bar = 100 μm. **(F)** Immunostaining and quantification of Islet-1 expression, which marks MNs, by cultures of iPSC-derived spinal neurons. Percentage of Islet-1 positive neurons were similar among the different genetic backgrounds. Scale bar = 100 μm. *n* = 3. Error bars indicate SEM. LL, long linker; SL, short linker; MUT, mutant; WT, wild type.

Mutations in FUS are associated with a particularly aggressive form of ALS, and insoluble FUS protein is found in a subset of sporadic ALS cases (Conlon et al., 2018). The most frequent ALS-associated *FUS* mutations are located in the nuclear localization signal, leading to its aberrant cytoplasmic accumulation, and MNs with the highest amounts of aberrant cytoplasmic FUS protein are those most likely to degenerate (Marrone et al., 2019). Previously, we established isogenic FUS-eGFP reporter iPSC lines (Marrone et al., 2018, 2019). In these cells, one allele of FUS is fused to eGFP via a linker peptide, which varies in length (Figure 1A). The other allele is unmodified and contains the WT *FUS* sequence. A long linker (LL) preserves the nuclear localization of the WT FUS-eGFP, whereas the FUS-P525L mutant variant, having this mutation in the FUS nuclear localization signal, induces its cytoplasmic mislocalization (Figures 1B,C). We previously demonstrated that cytoplasmic accumulation of FUS protein is heterogeneous in spinal MNs of *FUS*-ALS patients, with some showing severe cytoplasmic accumulation of FUS (Marrone et al., 2019). To model this more advanced pathogenesis, we previously generated and characterized FUS-eGFP reporter lines with a short linker (SL) disrupting the function of the adjacent nuclear localization signal and leading to increased cytoplasmic mislocalization of FUS (Figures 1A–C) (Marrone et al., 2018, 2019). The cell lines were differentiated into cultures composed almost entirely of neurons displaying the expected expression of multiple markers defining MNs as well as additional neuronal subtypes (Figures 1D,E). Eigengene classification identifies the cells in these cultures with whole spinal cord with 83% probability. All cell lines differentiated into Islet-1 positive MNs with a similar efficiency of approximately 50% (Figure 1F), and which we had previously demonstrated using this protocol as being electrophysiologically functional (Bursch et al., 2019). Since cultures expressed very high levels of spinal neuronal markers and were enriched with MNs, we hereafter refer to these cultures as iPSC-derived SNs.

### Modeling *FUS*-ALS Pathogenesis Using Monosynaptic Rabies Tracing

Neuronal connectivity is essential for function. Pathological ALS aggregates spread along neuronal circuits (Braak et al., 2013, 2017), and it has been shown that maintenance of MN connectivity is essential for preventing ALS phenotypes (Parone et al., 2013). Therefore, we used monosynaptic rabies tracing to assess neuronal connectivity of iPSC-derived neurons with either WT or P525L FUS-eGFP. To this aim, a lentiviral vector was used to overexpress the TVA Receptor and rabies glycoprotein, together with an iRFP reporter, in iPSC-derived spinal neuron progenitor cells, which are untargeted and therefore do not express the FUS-eGFP transgene. These cells were designated as “starter cells.”

**FIGURE 2:**
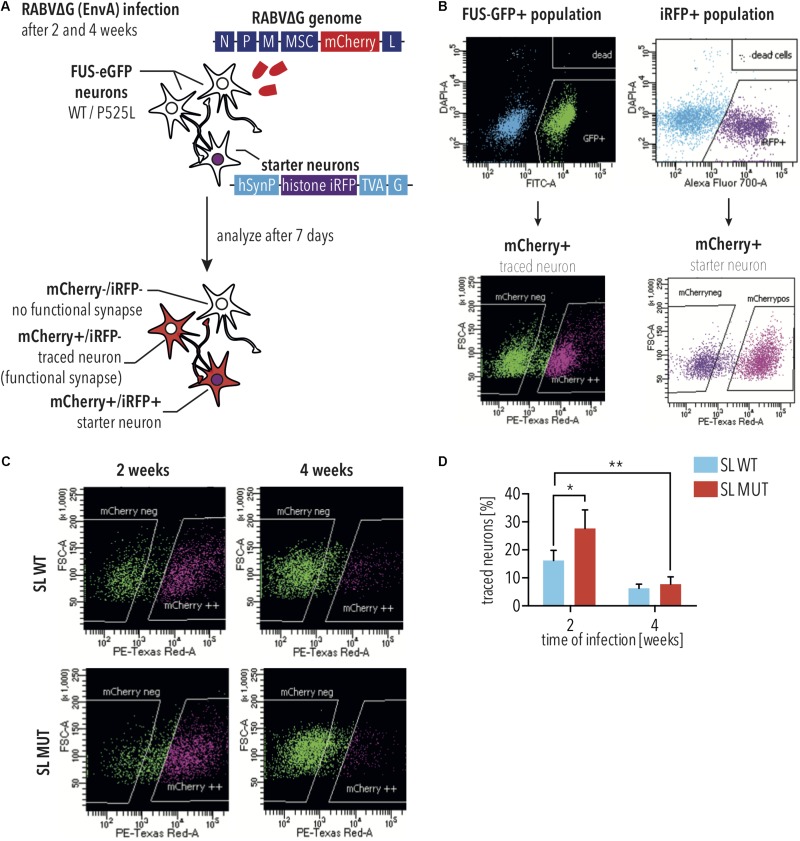
Monosynaptic tracing using RABVΔG(EnvA) identifies neurodegeneration in FUS-P525L spinal neurons. **(A)** Diagram illustrating monosynaptic tracing using RABVΔG(EnvA). Starter neurons express TVA-receptor, H2B-iRFP, and the rabies glycoprotein. Following infection with RABVΔG(EnvA), starter neurons also express mCherry. **(B)** Diagram illustrating analysis of monosynaptic tracing using flow cytometry. To quantify traced cells, cells are gated to select GFP- and mCherry-positive cells. To quantify starter cells, cells are gated to select iRFP- and mCherry-positive cells. **(C,D)** FUS-P525L neurons show lower number of traced cells at 4 weeks (*n* = 2) in comparison to earlier timepoints (*n* = 4). FUS-P525L neurons show higher mCherry expression when infected 2 weeks after seeding. Error bars indicate SEM. ^∗^ and ^∗∗^ correspond to *p* < 0.05 and 0.01, respectively. SL, short linker; MUT, mutant; WT, wild type; histone iRFP, H2B-iRFP.

To establish the disease model, we used iPSC-derived SNs with SL P525L FUS-eGFP due to these displaying the highest levels of mislocalized FUS, and would, therefore, provide the most severe phenotype. Neurons derived from starter cells were co-cultured with the SL FUS-eGFP lines (Figure 2A). EnvA pseudotyped rabies virus can only infect cells expressing the TVA Receptor. Thus, a reporter rabies vector, in which mCherry has replaced the rabies glycoprotein, was pseudotyped with a chimeric envelope protein consisting of the extracellular and transmembrane domains of EnvA fused to the cytoplasmic domain of the rabies virus glycoprotein [designated as RABVΔG(EnvA)]. RABVΔG(EnvA) was used to exclusively infect starter neurons expressing the TVA receptor (Figures 2A,B). Flow cytometry confirmed that RABVΔG(EnvA) only infected starter neurons (Supplementary Figure S1). To investigate age-dependent neurodegeneration, starter neurons were infected with RABVΔG(EnvA) at either 2 or 4 weeks following plating (Figure 2A). Co-cultures were analyzed using flow cytometry and number of traced cells were quantified (Figure 2D). Monosynaptic tracing resulted in mCherry-positive neurons and iRFP negative neurons. The number of traced iPSC-derived SNs with WT FUS-eGFP decreased less than 3-fold over time (not significant, 2way ANOVA, Sidak post-test for multiple comparisons; Figures 2C,D). In contrast, the number of traced iPSC-derived SNs with P525L FUS-eGFP showed a more than 3.6-fold decrease over time (*p* < 0.05, 2way ANOVA, Sidak post-test for multiple comparisons; Figures 2C,D), consistent with the age-dependent neurodegeneration observed in ALS.

Fused in sarcoma aggregation is a hallmark of FUS-ALS pathology, and it is believed that the recruitment of FUS to cytoplasmic SGs seeds its aggregation. To model spreading in culture, we used microfluidic chambers to separate the starter neuron from the traced FUS-eGFP population (Supplementary Figure S2A). Axons grew through the microchannels and formed neuronal circuits via synaptic connections. 2 and 4 weeks after plating in the microfluidic chambers, RABVΔG(EnvA) infected starter neurons expressing the TVA receptor, rabies glycoprotein and nuclear GFP. Cultures were imaged using confocal microscopy 7 days later (Supplementary Figures S2B,C). As expected, the percent of traced cells was noticeably decreased using the microfluidic system compared with bulk co-cultures (Supplementary Figures S2C,D). Consistent with our previous results, iPSC-derived SNs with WT *FUS* showed a trend toward decreased tracing over time, which did not reach statistical significance (Supplementary Figures S2C,D). iPSC-derived SNs with P525L FUS-eGFP showed a 5-fold decrease in tracing (*p* < 0.05, 2way ANOVA, Sidak post-test for multiple comparisons; Supplementary Figures S2C,D), consistent with the age-dependent neurodegeneration observed in ALS. Interestingly, traced cells, which were marked by RABVΔG-mCherry expression, often formed cytoplasmic foci containing FUS-eGFP, which is consistent with SG formation (arrowheads in Supplementary Figure S2E). This observation is important because it demonstrates that rabies tracing, which relies on the spreading of the virus, exacerbated two pathological hallmarks of *FUS*-ALS, including degeneration of iPSC-derived SNs as well as formation of FUS-positive cytoplasmic foci. It should be noted that spreading depends on axonal transport, and it has been shown that axonal transport is reduced in iPSC-derived ALS MNs (Guo et al., 2017; Naumann et al., 2018). Thus, the increased spreading of virus that we observed could be underestimated. Therefore, rabies tracing experiments using iPSC-derived SNs with mutant *FUS* are able to recapitulate time-dependent neurodegeneration, which is a hallmark of ALS pathogenesis.

### Rabies Virus Induces *FUS*-ALS Phenotypes in Spinal Neurons Due to Aberrant Defense

Although tracing experiments revealed that iPSC-derived SNs with SL P525L FUS-eGFP showed increased degeneration at 4 weeks compared with WT, we were surprised to consistently observe higher numbers of RABVΔG-mCherry-positive traced cells in P525L cultures compared with WT when infected for 2 weeks, representing the earliest time point measured in these experiments (Figure 2D and Supplementary Figure S2D). Since FUS was recently shown to act as a restriction factor against Kaposi’s Sarcoma-Associated Herpesvirus (Dunker et al., 2018), we hypothesized that this increased signal could indicate that the P525L mutation reduces the anti-viral defense activity of FUS protein, leading to increased RABVΔG-mCherry expression.

**FIGURE 3:**
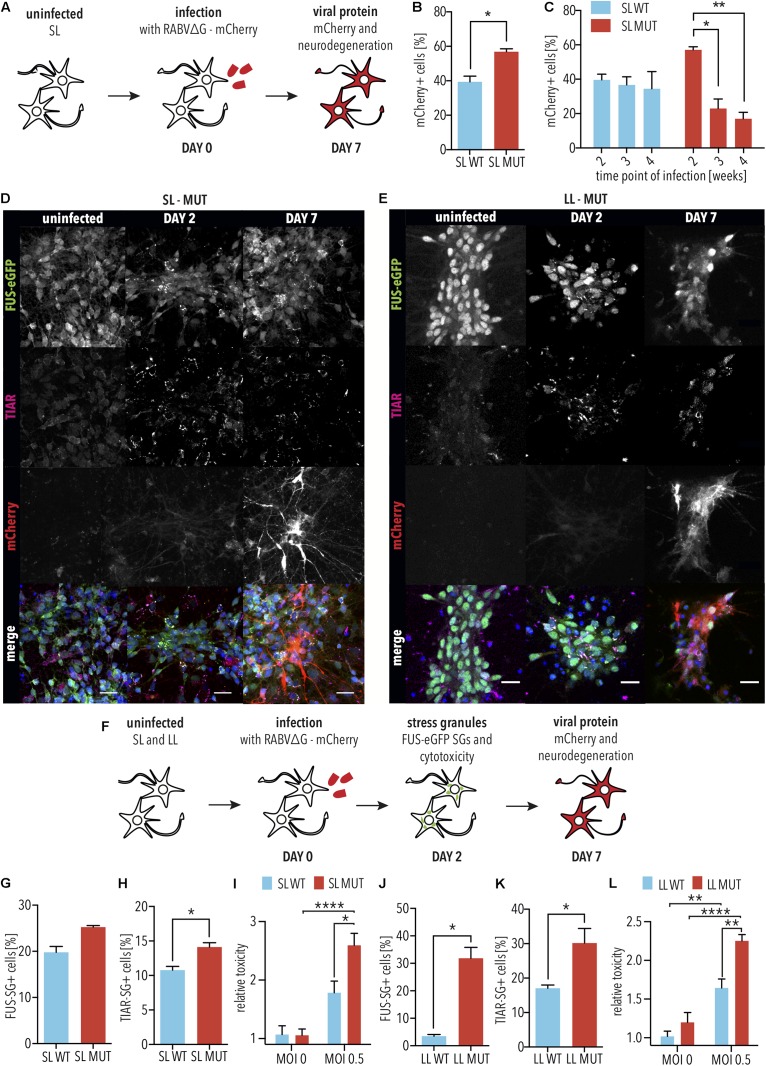
FUS-P525L spinal neurons show neurodegeneration due to altered virus defense against RABVΔG. **(A)** Diagram illustrating infection of iPSC-derived spinal neurons using RABVΔG. **(B)** SL FUS-P525L (SL MUT) show increased expression of RABVΔG-mCherry relative to WT (SL WT). 2 weeks after plating, neurons were infected with RABVΔG. Cultures were analyzed 7 days following infection. *n* = 3. Error bars indicate SEM. ^∗^ corresponds to *p* < 0.05 according to unpaired 2-tailed *t*-Test. **(C)** Number of RABVΔG-mCherry positive spinal neurons with the indicated genotype on the indicated time following infection are significantly lower indicating neurodegeneration. *n* = 3. Error bars indicate SEM. ^∗^ and ^∗∗^ correspond to *p* < 0.05 and 0.01, respectively, according to 2way ANOVA, Tukey post-test for multiple comparisons. **(D,E)** 2 days following infection, FUS-P525L spinal neurons show stress granules, and 7 days following infection, FUS-P525L spinal neurons show RABVΔG- mCherry transgene expression. Scale bar = 25 μm. **(F)** Diagram illustrating the time points for the quantification of the indicated ALS phenotypes following infection of iPSC-derived spinal neurons using RABVΔG. **(G–L)** Quantification of the FUS-eGFP-positive SGs **(G,J)**, TIAR-positive SGs **(H,K)**, and LDH release **(I,L)**, which measures cytotoxicity in spinal neurons with either SL FUS-eGFP **(G–I)** or LL FUS-eGFP **(J–L)** following infection with RABVΔG. ^∗^, ^∗∗^, and ^∗∗∗∗^ indicate *p* < 0.05, 0.01, and 0.0001, respectively, according to 2way ANOVA, Sidak post-test for multiple comparisons. *n* = 5. LL, long linker; SL, short linker; MUT, mutant; WT, wild type.

Since the cellular response to infection is highly dynamic, we first determined the time points for assessing RABVΔG-mCherry expression as well as ALS-associated phenotypes, including SG formation and neurodegeneration. For this, mCherry reporter RABVΔG was generated using the native glycoprotein (hereafter designated RABVΔG), which is able to infect iPSC-derived SNs independently of the EnvA receptor (Figure 3A). iPSC-derived SNs with SL P525L FUS-eGFP were infected with RABVΔG (Figures 3B–D). In order to determine whether differences would be due to the linker, we performed parallel analyses in iPSC-derived neurons with LL (Figure 3E). 2 days following infection, we observed the formation of TIAR-positive SGs (Figures 3D,E). RABVΔG-mCherry expression was observed 7 days following infection (Figures 3D,E).

Next, we sought to determine the effects of FUS P525L on RABVΔG-mCherry expression and SG formation. To do this, we assessed the levels of RABVΔG-mCherry expression in iPSC-derived SNs with P525L SL FUS-eGFP compared with WT at 7 days following infection with RABVΔG. We observed that iPSC-derived SNs with the P525L mutation showed significantly more RABVΔG-mCherry-positive neurons compared with WT (*p* < 0.05, unpaired 2-tailed *t*-Test; Figure 3B). WT SL FUS-eGFP showed no significant changes in the number of mCherry-positive neurons when infecting older cultures (3 or 4 weeks). In striking contrast, however, we found that P525L SL FUS-eGFP SNs showed significantly fewer RABVΔG-mCherry-positive with age (3 weeks: *p* < 0.05, 2way ANOVA, Tukey post-test for multiple comparisons; 4 weeks: *p* < 0.01, 2way ANOVA, Tukey post-test for multiple comparisons), suggesting accelerated neurodegeneration, consistent with age-dependent ALS pathogenesis (Figure 3C). Furthermore, it is interesting to note that the decreased number of traced cells could be due to increased degeneration of neurons as a result of mutant FUS or, possibly, that the virus is more toxic to neurons with mutant FUS.

Stress granules play a critical role in ALS as well as in defending cells against viruses, and we speculated that the differences in the increased number of RABVΔG-mCherry-positive SNs with mutant FUS relative to WT could be linked to aberrant SGs. We thus imaged SG marker TIAR and FUS-eGFP in neurons 2 days following infection with RABVΔG (Figures 3F–H and Supplementary Figure S3). iPSC-derived SNs with SL P525L FUS-eGFP showed increased numbers of FUS-eGFP positive SGs compared with WT (*p* = 0.13, unpaired 2-tailed *t*-Test; Figure 3G). Similarly, iPSC-derived SNs with SL P525L FUS-eGFP showed significantly increased numbers of TIAR-positive SGs compared with WT (*p* < 0.05, unpaired 2-tailed *t*-Test; Figure 3H). Similar results were obtained with iPSC-derived SNs with LL P525L FUS-eGFP (Figures 3J,K). Therefore, the increased levels of RABVΔG-mCherry in iPSC-derived SNs with mutant FUS correlate with the formation of aberrant SGs, which show increased recruitment of FUS protein, and which is linked to ALS pathogenesis.

The increased viral transgene expression, together with the formation of aberrant SGs, led us to speculate that the survival of P525L FUS SNs from RABVΔG infection would be decreased relative to WT. Spinal neurons from both sets of isogenic lines were differentiated and infected with RABVΔG. After 2 days, LDH release was used to quantify degeneration. As expected, we observed that SNs with SL P525L FUS-eGFP showed significantly increased degeneration compared with WT after RABVΔG infection (*p* < 0.05, 2way ANOVA, Sidak post-test for multiple comparisons; Figure 3I). Similar results were obtained using iPSC-derived SNs with LL P525L FUS-eGFP (*p* < 0.01, 2way ANOVA, Sidak post-test for multiple comparisons; Figure 3L).

Proteasomal dysfunction plays a critical role in ALS (Shahheydari et al., 2017), and we demonstrated that mutant FUS induced defects in protein homeostasis in iPSC-derived SNs (Marrone et al., 2018, 2019), leading us to speculate that proteasomal defects might contribute to the sensitivity of SNs with mutant FUS to rabies infection. To test this, SNs with LL WT FUS-eGFP, in which FUS localization is exclusively nuclear, were infected with RABVΔG in the presence or absence of proteasome inhibitor MG132. 2 days following infection, when RABVΔG-mCherry levels were relatively low in the absence of MG132 (Figure 3B as well as Supplementary Figures S4A,B), we observed that SNs treated with MG132 showed significantly increased levels of RABVΔG-mCherry (*p* < 0.05, unpaired 2-tailed *t*-Test; Supplementary Figure S4C). Immunostaining for the apoptosis marker, cleaved-Caspase 3 (CC3), demonstrated that SNs treated with MG132 as well as RABVΔG showed significantly higher levels of CC3 compared with RABVΔG alone (*p* < 0.01, unpaired 2-tailed *t*-Test; Supplementary Figures S4D,E).

Taken together, these results demonstrate that RABVΔG infection exacerbated the formation of aberrant SGs as well as the degeneration of SNs with mutant FUS, hallmarks of *FUS*-ALS pathogenesis. These results could suggest that, in some patients, viral infections may play a critical role in ALS pathogenesis by exacerbating the effects of inherited mutations. The spreading of viruses along neuronal circuits represents a possible mechanism by which some viruses facilitate the prion-like spreading of ALS pathogenesis.

### HIV-1 and ZIKV Exacerbate Degeneration of Spinal Neurons With Mutant FUS Independently of SG Formation

**FIGURE 4:**
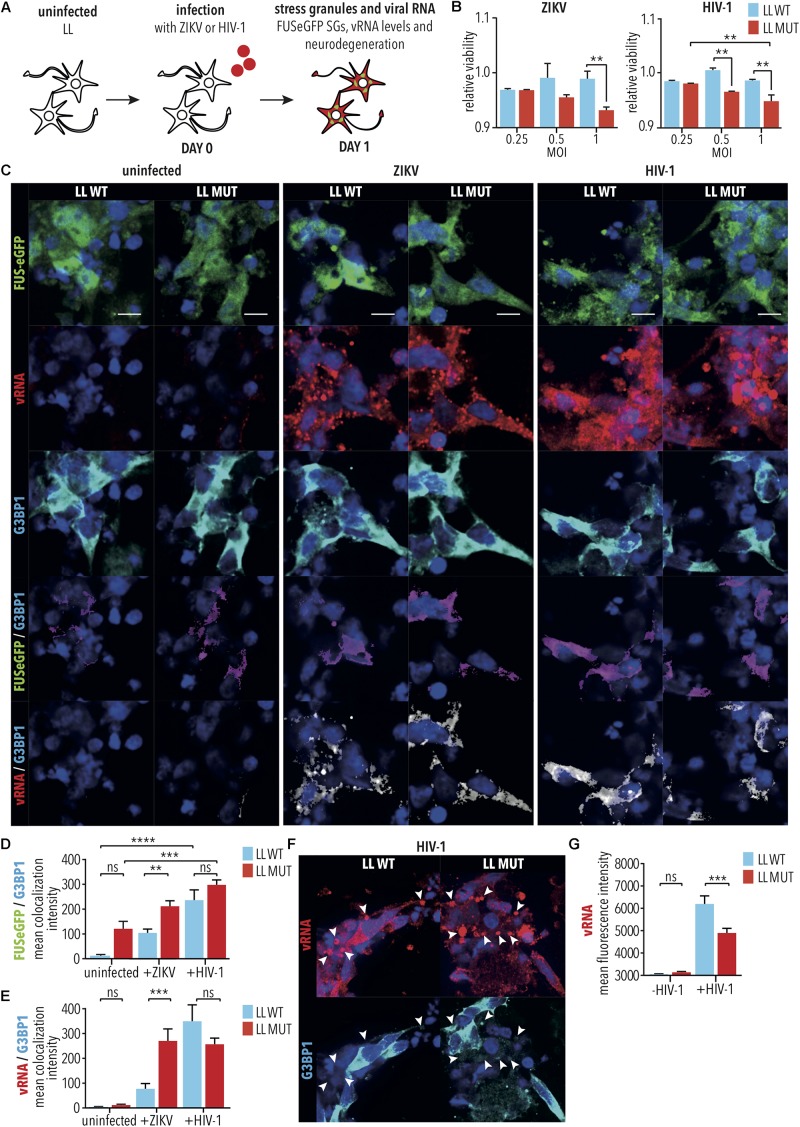
Infection of iPSC-derived spinal neurons with HIV-1 and ZIKV. **(A)** Diagram illustrating HIV-1 or ZIKV infection of iPSC-derived spinal neurons with either WT FUS (LL WT) or FUS-P525L (LL MUT). **(B)** MTT assay was used to quantify viability of spinal neurons 1 day following infection under the indicated conditions. ^∗∗^ indicates *p* < 0.01 according to 2way ANOVA, Tukey post-test for multiple comparisons. *n* = 3. **(C)** Spinal neurons treated as indicated followed by imaging for vRNA as well as SG marker G3BP1. Scale bar = 5 μm. **(D,E)** Co-localization analyses between **(D)** G3BP1 and FUS, **(E)** G3BP1 and vRNA following infection of WT and FUS-P525L neurons. ^∗∗^, ^∗∗∗^, and ^∗∗∗∗^ indicate *p* < 0.01, 0.001, and 0.0001, respectively, according to 2way ANOVA, Tukey post-test for multiple comparisons. Error bars represent SEM. *n* = 6. **(F)** HIV-1 vRNA appeared to accumulate in large G3BP1-negative cytoplasmic bodies (arrow heads). **(G)** HIV-1 and ZIKV vRNA levels following infection of WT and mutated FUS-P525L neurons. ^∗∗∗^ indicates *p* < 0.001, according to 2way ANOVA, Tukey post-test for multiple comparisons. Error bars represent SEM. *n* = 6. MOI indicates multiplicity of infection; SG, stress granule; LL, long linker; MUT, mutant; WT, wild type; vRNA, viral RNA; /, colocalization.

Amyotrophic lateral sclerosis cases have been associated with concurrent HIV-1 infection (Alfahad and Nath, 2013), and clinical ALS phenotypes of some HIV-1 patients have been shown to be ameliorated through administration of anti-viral drugs (Moulignier et al., 2001), suggesting that HIV-1 infection contributes to ALS pathogenesis. Similarly, infection by ZIKV has been demonstrated to cause MN degeneration (Ramalho et al., 2017; Zukor et al., 2018). We would, therefore, expect that HIV-1 and ZIKV infection would exacerbate degeneration of iPSC-derived SNs with mutant *FUS*. To test this, differentiated SNs with LL WT or P525L FUS-eGFP were infected with either HIV-1 (i.e., HIV-1 pseudotyped with VSVG-env to increase infection of SNs) or ZIKV [i.e., Canadian imported Thai ZIKV strain PLCal_ZV (Alpuche-Lazcano et al., 2018)] at increasing multiplicities of infection (MOI) of 0–1 for 2 h, prior to collecting cells 24 h later for quantification of ensuing cell viability using a colorimetric MTT assay measuring cell metabolic activity (Figure 4A). Consistent with our previous results from RABV infection (Figures 3H,K), P525L LL FUS-eGFP SNs showed reduced viability with increasing MOI for both HIV-1 (MOI 1, LL MUT vs. LL WT, *p* < 0.01, 2way ANOVA, Tukey post-test for multiple comparisons) and ZIKV (MOI 1, LL MUT vs. LL WT, *p* < 0.01, 2way ANOVA, Tukey post-test for multiple comparisons), and thus increased degeneration compared with WT FUS after infection by either HIV-1 or ZIKV (Figure 4B). The effects of increasing MOI on cell viability was especially significant for the P525L FUS-eGFP SNs (LL MUT, MOI 0.25 vs. 1, *p* < 0.01, 2-way ANOVA, Tukey post-test for multiple comparisons), whereas the viability of WT FUS neurons was not significantly affected by infection with HIV-1 or ZIKV (LL WT, MOI 0.25 vs. 1, HIV-1: not significant, 2way ANOVA, Tukey post-test for multiple comparisons, ZIKV: not significant, 2way ANOVA, Tukey post-test for multiple comparisons; Figure 4B).

Aberrant SG formation is believed to play an important role in ALS pathogenesis (Li et al., 2013). Since HIV-1 and ZIKV are both well documented to inhibit SG assembly in order to facilitate viral replication (Valiente-Echeverria et al., 2014; Amorim et al., 2017), it was surprising to observe that, like RABV, HIV-1, and ZIKV also exacerbated the degeneration of SNs with mutant *FUS*. This suggests that either the molecular mechanism of viral replication is altered in iPSC-derived SNs relative to other cell types, or, alternatively, that additional virus-associated mechanisms exacerbate neurodegeneration. To better understand the contribution of SGs to neurodegeneration induced by HIV-1 and ZIKV, similar experiments were carried out on neurons for imaging (Figure 4A), where HIV-1 and ZIKV infected spinal neuron cultures were fixed for immunostaining for G3BP1, FUS-eGFP and viral RNA. G3BP1 and TIAR are both stress granule markers and are often used interchangeably to accommodate for differences in host species producing antibodies while assembling multi-color immunofluorescence panels. As expected, we saw no evidence of G3BP1-positive SG formation under any condition tested (Figures 4C,D), despite our demonstration that these cells were capable of producing G3BP1-positive SGs when applying arsenite (Supplementary Figure S5). Quantification of protein colocalization demonstrated that, despite lack of SG formation, G3BP1/FUS colocalization was increased in both WT FUS and FUS-P525L mutant SNs following virus infection by either HIV-1 (e.g., LL MUT, -virus vs. HIV-1, *p* < 0.001, 1way ANOVA, Tukey post-test for multiple comparisons), or ZIKV (e.g., ZIKV, LL WT vs. LL MUT, *p* < 0.01, 1way ANOVA, Tukey post-test for multiple comparisons; Figures 4C,D). G3BP1 colocalization with the ZIKV viral RNA (vRNA) was also increased in the P525L FUS relative to the WT FUS SNs (ZIKV, LL WT vs. LL MUT, *p* < 0.001, 1way ANOVA, Tukey post-test for multiple comparisons; Figures 4C,E), suggesting that, despite ZIKV preventing the formation of SGs, SNs with mutant FUS were more sensitive to, and less able to control, the cellular stress induced by the infection. Colocalization of G3BP1 with the HIV-1 vRNA was not observed to be significant between WT and mutant FUS, however, the vRNA appeared to accumulate in large G3BP1-negative cytoplasmic bodies measuring 1–3 μm [HIV-1, LL WT vs. LL MUT, not significant, 1way ANOVA, Tukey post-test for multiple comparisons; Figures 4C,E,F(arrow heads)]. This could signify a disruption in the later stages of HIV-1 replication, potentially because SNs are not natural hosts for HIV-1 infection. The observation of these large HIV-1 vRNA cytoplasmic bodies may also be a strong indicator of the cytotoxicity of the infection to the cells, more highly affected in the FUS-P525L mutant (Figures 4C,G), and also possibly reflected by the observation of lowered HIV-1 vRNA expression in this more sensitive spinal neuron variant strain, relative to WT FUS (HIV-1, LL WT vs. LL MUT, *p* < 0.001, 1way ANOVA, Tukey post-test for multiple comparisons; Figures 4F,G). Following viral infection, cultures of iPSC-derived SNs were a heterogeneous mixture of viable and degenerating neurons. Since we speculated that vRNA levels could vary between neurons, particularly during degeneration, we manually selected viable neurons that showed high levels of cytoplasmic FUS and G3BP1. We found increased vRNA levels in P525L FUS compared with WT FUS control neurons (Supplementary Figure S6). This suggests that HIV-1 vRNA cytoplasmic bodies lacking G3BP1 might result from neurodegeneration induced by elevated replication of HIV-1 RNA.

Altogether, these results support the hypothesis that SNs with mutant FUS are under stress from the ALS mutation, and the addition of a viral infection increases the risk of degeneration compared with WT. Since rabies virus, HIV-1, and ZIKV are significantly different from each other, our results demonstrate that iPSC-derived SNs with mutant FUS are more sensitive to degeneration after viral infection irrespective of the specific type of virus. This suggests a synergistic cytotoxic effect of mutated FUS and virus infection on spinal neuron pathophysiology.

### RABV and HIV-1 Exacerbate Cytoplasmic Mislocalization of FUS Protein

**FIGURE 5:**
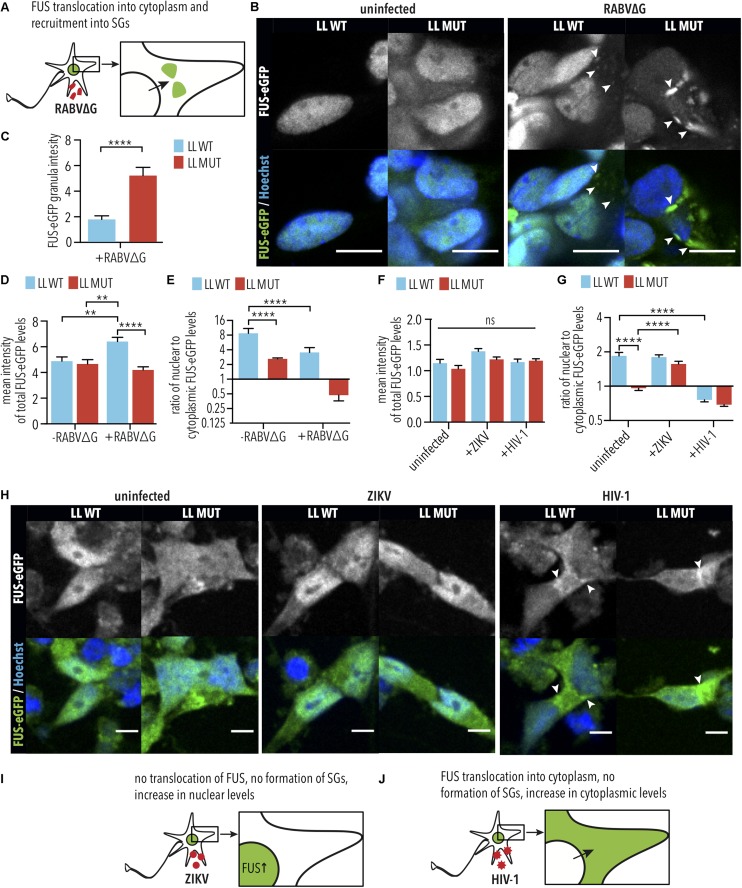
RABVΔG and HIV-1 induce FUS-eGFP nuclear to cytoplasmic translocation. **(A)** Schematic view of changes in FUS-eGFP during RABVΔG infection. **(B)** FUS-P525L spinal neurons show increased translocation of FUS-eGFP from the nucleus to the cytoplasm as well as recruitment to SGs. Mean FUS-eGFP intensity was measured from confocal images. White arrows indicate SGs. Scale bar = 10 μm. **(C)** FUS-eGFP levels in RABVΔG-induced granules are increased in FUS-P525L neurons in comparison to WT. ^∗∗∗∗^ indicate *p* < 0.0001, according to 2-tailed unpaired *t-*test. Error bars represent SEM. *n* = 3. **(D)** FUS-WT spinal neurons infected with RABVΔG show increased total FUS-eGFP mean intensity levels. ^∗∗^ and ^∗∗∗∗^ indicate *p* < 0.01 and 0.0001, respectively. *n* = 3. **(E)** FUS-eGFP is mislocalized following RABVΔG infection of spinal neurons. Predominant localization of FUS-eGFP is nuclear if ratio >1, cytoplasmic (mislocalized) if ratio <1. ^∗∗∗∗^ indicates *p* < 0.0001. Error bars represent SEM. **(F)** Total FUS-eGFP levels were unaffected by HIV-1 and ZIKV infection. ns, Not significant. *n* = 6. **(G)** FUS-eGFP is mislocalized in FUS-P525L and WT HIV-1 infected spinal neurons. ^∗∗∗∗^ indicates *p* < 0.0001 according to one-way ANOVA, Tukey post-test for multiple comparisons. Error bars represent SEM. **(H)** FUS-P525L and FUS-WT spinal neurons show increased translocation of FUS-eGFP out of the nucleus, into the cytoplasm after HIV-1 infection. White arrows indicate increased FUS-eGFP levels at nuclear periphery. Scale bar = 10 μm. **(I,J)** Schematic view of changes of FUS-eGFP during ZIKV and HIV-1 infection. LL indicates long linker; MUT, mutant; WT, wild type; vRNA, viral RNA; SG, stress granules.

It was previously demonstrated that HIV-1 induces cytoplasmic retention of hnRNPA1 (Monette et al., 2009), which is an RBP that is structurally similar to FUS and is also associated with ALS (Kim et al., 2013). Therefore, we hypothesized that viral infections might similarly induce cytoplasmic retention of FUS, which could exacerbate the effects of the P525L mutation. To test this, we quantified the nuclear and cytoplasmic levels of WT and P525L LL FUS-eGFP in iPSC-derived SNs following infection with RABVΔG, HIV, or ZIKV (Figure 5). As expected, iPSC-derived SNs expressing LL P525L FUS-eGFP showed significantly increased levels of FUS-eGFP in SGs (arrow heads in Figure 5B) following RABVΔG infection compared with WT (LL MUT uninfected vs. LL MUT infected, *p* < 0.0001, 1way ANOVA, Tukey post-test for multiple comparisons; Figures 5B,C). We observed that iPSC-derived SNs with WT FUS-eGFP showed increased total FUS-eGFP levels (cytoplasmic + nuclear FUS-eGFP) following RABVΔG infection compared with uninfected controls (RABVΔG total: LL WT uninfected vs. LL WT infected, *p* < 0.01, 1way ANOVA, Tukey post-test for multiple comparisons; Figure 5D), suggesting that RABVΔG infection caused defects in FUS turnover. In addition, RABVΔG infection induced significant reduction in the nuclear to cytoplasmic ratio of LL WT FUS-eGFP in iPSC-derived SNs and increased the mislocalization of LL P525L FUS-eGFP (LL MUT uninfected vs. LL MUT infected, not significant, 1way ANOVA, Tukey post-test for multiple comparisons; LL WT uninfected vs. LL WT infected, *p* < 0.0001, 1way ANOVA, Tukey post-test for multiple comparisons; Figure 5E).

Similar results were obtained using iPSC-derived SNs following infection with HIV-1, where increases in cytoplasmic FUS-eGFP was observed for both WT FUS and P525L FUS (arrow heads in Figure 5H). However, no differences were observed in total FUS-eGFP levels (Figure 5F), suggesting that HIV-1 infection induces cytoplasmic mislocalization without affecting FUS turnover. Consistent with this, HIV-1 infection induced significant reduction in the nuclear to cytoplasmic ratio of LL WT FUS-eGFP in iPSC-derived SNs and increased the mislocalization of LL P525L FUS-eGFP (LL MUT uninfected vs. LL MUT infected, not significant, 1way ANOVA, Tukey post-test for multiple comparisons; LL WT uninfected vs. LL WT infected, *p* < 0.0001, 1way ANOVA, Tukey post-test for multiple comparisons; Figure 5G). This finding is particularly interesting because cytoplasmic FUS mislocalization is one of the molecular events driving *FUS*-ALS.

In contrast to RABV and HIV-1, ZIKV infection did not induce cytoplasmic FUS accumulation in iPSC-derived SNs harboring either WT and P525L LL FUS-eGFP (Figure 5H). Surprisingly, we found that ZIKV infection increased nuclear to cytoplasmic levels of P525L FUS-eGFP relative to uninfected controls (LL MUT uninfected vs. LL MUT infected, *p* < 0.0001, 1way ANOVA, Tukey post-test for multiple comparisons, Figures 5G,H). Similar to HIV-1, no difference was observed in total WT or P525L FUS-eGFP levels following infection with ZIKV (Figure 5E). These data demonstrate that there are virus species-specific interactions with ALS genetic risk factors. Our results demonstrate that iPSC-derived SNs are effective tools for characterizing these interactions, which, in the future, could lead to improved diagnostics tools as well as therapeutic strategies for ALS patients.

## Discussion

The majority of ALS cases are sporadic and result from a poorly understood interaction between genetic polymorphisms, such as FUS P525L (Conte et al., 2012), and environmental risk factors, in which specific viruses such as HIV-1 have been implicated (Moulignier et al., 2001). A hallmark of ALS pathogenesis is the degeneration of SNs, which causes progressive paralysis in patients, and we are the first to report that viral infections, including with RABV, ZIKV, and HIV-1 exacerbate the degeneration of iPSC-derived SNs with FUS P525L.

Similar cellular pathways are commonly shared between ALS and viral pathogenesis, suggesting that there are multiple molecular mechanisms by which viral infections may exacerbate the effects of genetic ALS risk factors. For example, ALS is often characterized by the cytoplasmic mislocalization of specific RBPs, including FUS. We demonstrate that this mislocalization is exacerbated by specific viruses, including RABV and HIV-1. In addition, it is important to note that this effect is not specific to FUS. It has been previously demonstrated that HIV-1 infection induces cytoplasmic retention of RBPs harboring prion-like domains, including hnRNPA1 and hnRNPD (Monette et al., 2009; Lund et al., 2012). A proteomic study characterizing ribonucleoprotein complexes involved in HIV-1 assembly in the cytoplasm suggests that many more RBPs containing prion-like domains could be mislocalized following HIV-1 infection, including TDP-43 (Milev et al., 2012). Interestingly, it has been shown that cytoplasmic mislocalization of ALS-associated RBPs interferes with intra-axonal protein translation, leading to neurodegeneration even in the absence of aggregate formation (Lopez-Erauskin et al., 2018). Thus, our results suggest that virus-induced RBP mislocalization in SNs could lead to ALS pathology and the development of ALS-like symptoms. Similarly, it has been reported that VSV and EV, which are ssRNA viruses similar to HIV-1 and RABV, induce cytoplasmic retention of RBPs harboring prion-like domains, including hnRNPA1 and TDP-43 (Pettit Kneller et al., 2009; Xue et al., 2018). However, it is significant to note that not all viruses induce cytoplasmic retention of FUS. For example, ZIKV showed no effects on FUS mislocalization. Our iPSC-based platform could be a useful tool for evaluating the ability of specific viruses to exacerbate the mislocalization of ALS-associated RBPs.

One of the characteristics of ALS is that aggregates of RBPs tend to spread along neuronal circuitry in a corticofugal manner (Braak et al., 2013, 2017). We suggest that viruses could significantly exacerbate the spreading of ALS pathology. For example, viruses such as RABV have the ability to spread along neuronal circuits, which has led to development of monosynaptic tracing (Wickersham et al., 2007). In addition, we have demonstrated that viruses such as RABV increase cytoplasmic FUS levels, induce the formation of aberrant SGs containing mutant FUS, and increase neurodegeneration, which likely results in the release of extracellular vesicles that contain pathogenic FUS, thus facilitating prion-like spreading. Moreover, we showed that ALS mutations in FUS reduce their viral restriction activity, potentially leading to increased spreading of ALS phenotypes.

Although cytoplasmic FUS mislocalization as well as the formation of aberrant SGs play important roles in *FUS*-ALS (Li et al., 2013), our results demonstrate that it is possible for viruses such as ZIKV to exacerbate neurodegeneration without affecting these processes. This is important because it suggests that there are additional molecular mechanisms by which viral infections could interact with ALS pathogenesis. Previously, iPSC-derived SNs with mutant *FUS* were shown to be more likely to degenerate compared with isogenic WT controls as a result of disruption in the levels of multiple ALS-associated RNA-binding proteins, including hnRNPA1, hnRNPA2, EWSR1, and TAF15 (Marrone et al., 2018). The further disrupting of any of these proteins by a viral infection would lead to increased neurodegeneration. Another possibility is that neurons with mutant *FUS* are already under significant stress due to the disruption of many RNA-binding proteins, suggesting that an additional stress such as viral infection is sufficient to unmask ALS phenotypes in SNs with genetic ALS mutations.

Although specific ALS cases have been associated with HIV-1 infection (Alfahad and Nath, 2013), most patients with HIV infections do not develop ALS phenotypes. Since about 60% of the risk of sporadic ALS is inherited (Graham et al., 1997; Al-Chalabi et al., 2010), we suggest that this could be due to the low prevalence of genetic ALS risk factors. It has been shown that macrophages and microglial cells in the central nervous system are targets of HIV-1 infection (Vazeux et al., 1987). Since ALS-associated RBPs such as FUS and TDP-43 are ubiquitously expressed, it is conceivable that viral infections in immune cells could exacerbate ALS pathogenesis and spread to neurons via prion-like spreading. However, it is intriguing to note that it has been previously reported that HIV-1 can infect neurons (Canto-Nogues et al., 2005), and HIV-1 genes and proteins have been reported as observed in neurons of patients (Nuovo et al., 1994; Bagasra et al., 1996), suggesting that there could be a direct interaction in SNs between HIV-1 infection and genetic ALS risk factors.

Our data demonstrate that specific viruses such as RABV and HIV-1 exacerbate ALS phenotypes in iPSC-derived SNs with genetic risk factors such as FUS P525L, leading to increased cytoplasmic accumulation and neurodegeneration. Understanding the molecular mechanism of this interaction could improve the ability to identify individuals at risk of developing sporadic ALS. For example, in rare cases, it has been possible to reverse ALS-like symptoms in ALS cases linked to HIV infection (Alfahad and Nath, 2013). Using our iPSC-based approach, other viruses, such as ssRNA EV, could be investigated for their ability to exacerbate ALS phenotypes in SNs with ALS risk alleles as well as provide evidence that specific viruses may contribute to the onset of ALS disease in a specific genetic context. These viruses could be further investigated using epidemiology and potentially classified as risk factors for carriers of specific genetic risk factors, potentially enabling the development of personalized treatments, which could include specific anti-viral drugs, to prevent the onset of ALS in specific cases.

## Data Availability Statement

All datasets generated for this study are included in the manuscript/Supplementary Files.

## Author Contributions

JB, AM, AJM, MB, and JS designed the work and drafted the manuscript. JB, VT, AS, AM, MA-R, AJ, RB, and MB performed the experiments and analyzed the data. JB, AM, AJM, and JS reviewed and edited the manuscript. All authors have approved the final version of the manuscript and have agreed to be accountable for all aspects of the work regarding questions related to the accuracy or integrity of any part of the work.

## Conflict of Interest

RB is an employee of Verge Genomics. The remaining authors declare that the research was conducted in the absence of any commercial or financial relationships that could be construed as a potential conflict of interest.

## Footnotes

1 http://asia.ensembl.org/index.html

## Supplementary Material

FIGURE S1 RABVΔG(EnvA) specifically infects starter neurons expressing the TVA receptor. **(A)** 293T cells expressing the TVA receptor can be infected with RABVΔG(EnvA), visualized by mCherry expression. 293T cells lacking the TVA receptor cannot be infected by RABVΔG(EnvA), as shown by the lack of mCherry expression. **(B)** Flow cytometric analysis showing that TVA receptor-negative, which are GFP-negative, and mCherry-positive neurons, which mark traced neurons, can only be detected when co-culturing starter neurons, which are GFP-positive. **(C)** WT and P525L FUS-eGFP spinal neurons are not infected with RABVΔG(EnvA) because they do not express the TVA receptor. Flow cytometric analysis of infected FUS-eGFP neurons showed no differences increase in mCherry levels compared with uninfected neurons. Starter neurons, which express iRFP, TVA receptor, and rabies glycoprotein, have increased mCherry levels following infection with RABVΔG(EnvA) comparison with uninfected starter neurons.

FIGURE S2 RABVΔG(EnvA) tracing exacerbated two pathological hallmarks of *FUS*-ALS. **(A)** Diagram illustrating monosynaptic tracing using RABVΔG(EnvA) and microfluidic chambers. **(B)** Starter neurons express the TVA receptor, H2B-GFP and rabies glycoprotein. Following infection with RABVΔG(EnvA), starter neurons also express mCherry. **(C,D)** P525L SL FUS-eGFP neurons show fewer traced cells 4 weeks compared with 2 weeks following infection as marked by RABVΔG(EnvA)-mCherry transgene expression. *n* = 2. Error bars indicate SEM. ^∗^ and ^∗∗^ correspond to *p* < 0.05 and 0.01, respectively, according to 2way ANOVA, Sidak post-test for multiple comparisons. **(E)** Traced neurons show FUS-eGFP granules. White arrows indicate neurons positive for stress granules and mCherry transgene expression. SL, short linker; MUT, mutant; WT, wild type; histone GFP, H2B-GFP.

FIGURE S3 Direkt infection of LL and SL FUS-eGFP WT spinal neurons with RABVΔG-mCherry. 2 days following infection, FUS-WT spinal neurons show stress granules, and 7 days following infection, FUS-WT spinal neurons show RABVΔG-mCherry transgene expression. Scale bar = 25 μm.

FIGURE S4 Proteasomal inhibition increases RABVΔG-mCherry levels and neurodegeneration. **(A)** Diagram illustrating infection of iPSC-derived spinal neurons with RABVΔG in the presence of 2.5 μM MG-132. **(B,C)** RABVΔG-mCherry levels are increased in iPSC-derived spinal neurons with LL WT FUS-eGFP at 2 days following infection of spinal neurons in presence of the proteasome inhibitor MG-132. **(D,E)** Cleaved-Caspase3 (CC3) levels are increased in iPSC-derived spinal neurons with LL WT FUS-eGFP at 2 days following infection of spinal neurons in presence of the proteasome inhibitor MG-132. Scale bar = 25 μm. *n* = 3. Error bars indicate SEM. ^∗^ and ^∗∗^ correspond to *p* < 0.05 and 0.01, respectively. LL, long linker; MUT, mutant; WT, wild type.

FIGURE S5 FUS-eGFP-positive SGs form following arsenite treatment but not HIV-1 or ZIKV infection. **(A)** J2 antibody was validated in Vero cells infected with the same viral stocks used in MNs. ZIKV infected Vero cells show colocalization of vRNA, capsid (CA) and TIAR at the nuclear periphery. Scale bar = 10 μm. **(B)** FUS-eGFP spinal neurons show FUS-eGFP and G3BP1 positive SGs 1 h after adding 500 μM arsenite. Scale bar = 5 μm. **(C)** Spinal neurons do not show FUS-eGFP granule formation after infection. The G3BP1 and vRNA in ZIKV infected WT-FUS cell colocalizes in cytoplasmic puctae. Co-localized G3BP1 and vRNA, are much more diffuse in the P525L FUS mutant infected by both ZIKV or HIV-1, as it is for HIV-1 infected FUS WT cells. Scale bar = 5 μm. MOI, multiplicity of infection; DIC, differential interference contrast; CA, ZIKV capsid; LL, long linker; MUT, mutant; WT, wild type; vRNA, viral RNA; /, colocalization.

FIGURE S6 FUS-P525L spinal neurons show increased cytoplasmic vRNA levels **(A,B)** vRNA levels in FUS-P525L spinal neurons are increased in the cytoplasmic localization at the nuclear periphery after ZIKV or HIV-1 infection in comparison to WT. Scale bar = 10 μm. ^∗^ and ^∗∗^ indicate *p* < 0.01 and 0.0001, respectively, according to one-way ANOVA, Tukey post-test for multiple comparisons. *n* = 6. Error bars represent SEM.
