# Supplementary figures and images for: Viral Infections Exacerbate FUS-ALS Phenotypes in iPSC-Derived Spinal Neurons in a Virus Species-Specific Manner
Source: Front Cell Neurosci. 2019 Oct 22;13:480. doi: 10.3389/fncel.2019.00480 (PMC6817715; doi:10.3389/fncel.2019.00480)

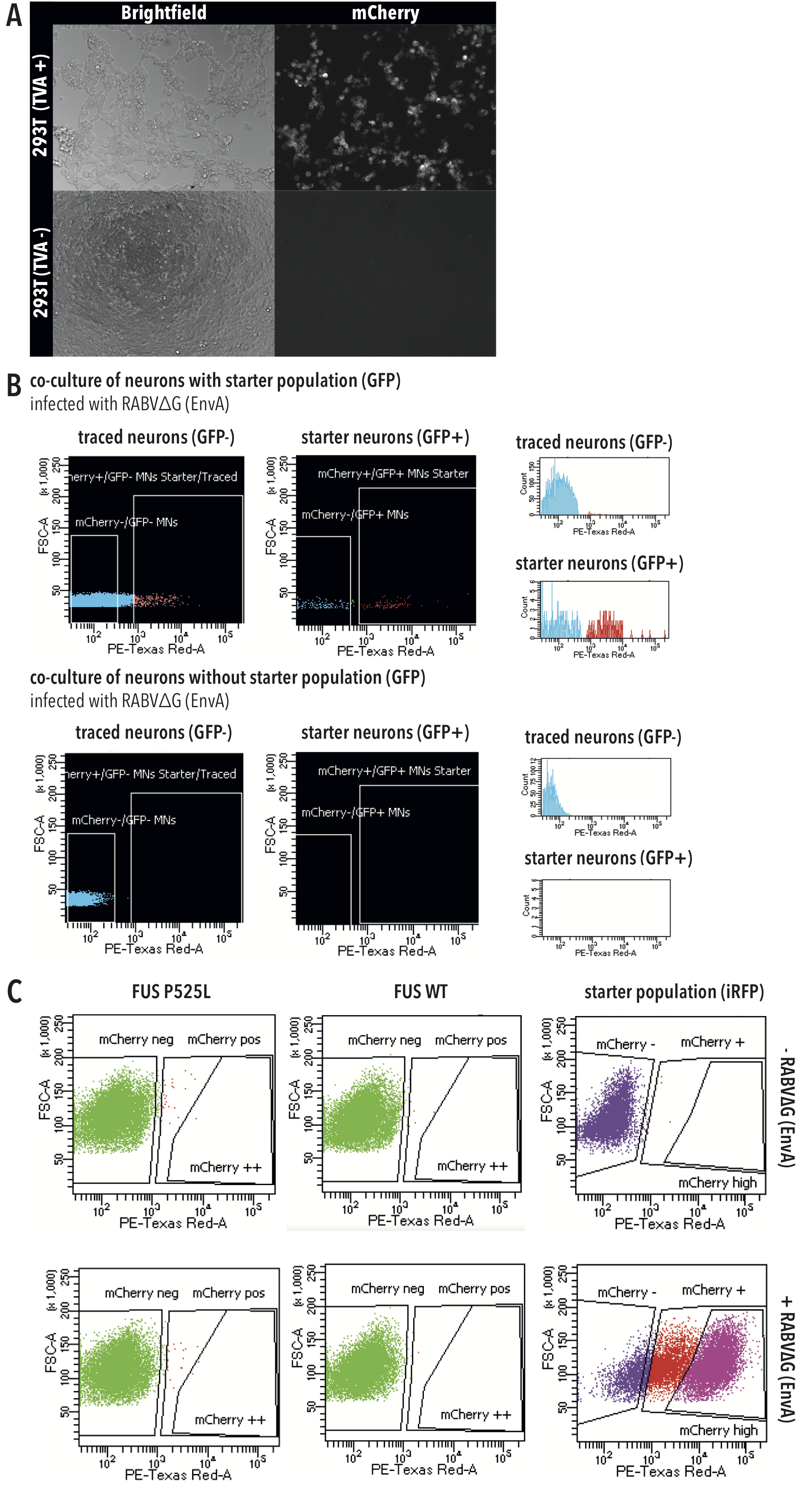

Supplement: FIGURE S1 — RABVΔG(EnvA) specifically infects starter neurons expressing the TVA receptor. (A) 293T cells expressing the TVA receptor can be infected with RABVΔG(EnvA), visualized by mCherry expression. 293T cells lacking the TVA receptor cannot be infected by RABVΔG(EnvA), as shown by the lack of mCherry expression. (B) Flow cytometric analysis showing that TVA receptor-negative, which are GFP-negative, and mCherry-positive neurons, which mark traced neurons, can only be detected when co-culturing starter neurons, which are GFP-positive. (C) WT and P525L FUS-eGFP spinal neurons are not infected with RABVΔG(EnvA) because they do not express the TVA receptor. Flow cytometric analysis of infected FUS-eGFP neurons showed no differences increase in mCherry levels compared with uninfected neurons. Starter neurons, which express iRFP, TVA receptor, and rabies glycoprotein, have increased mCherry levels following infection with RABVΔG(EnvA) comparison with uninfected starter neurons. [file Image_1.TIFF]

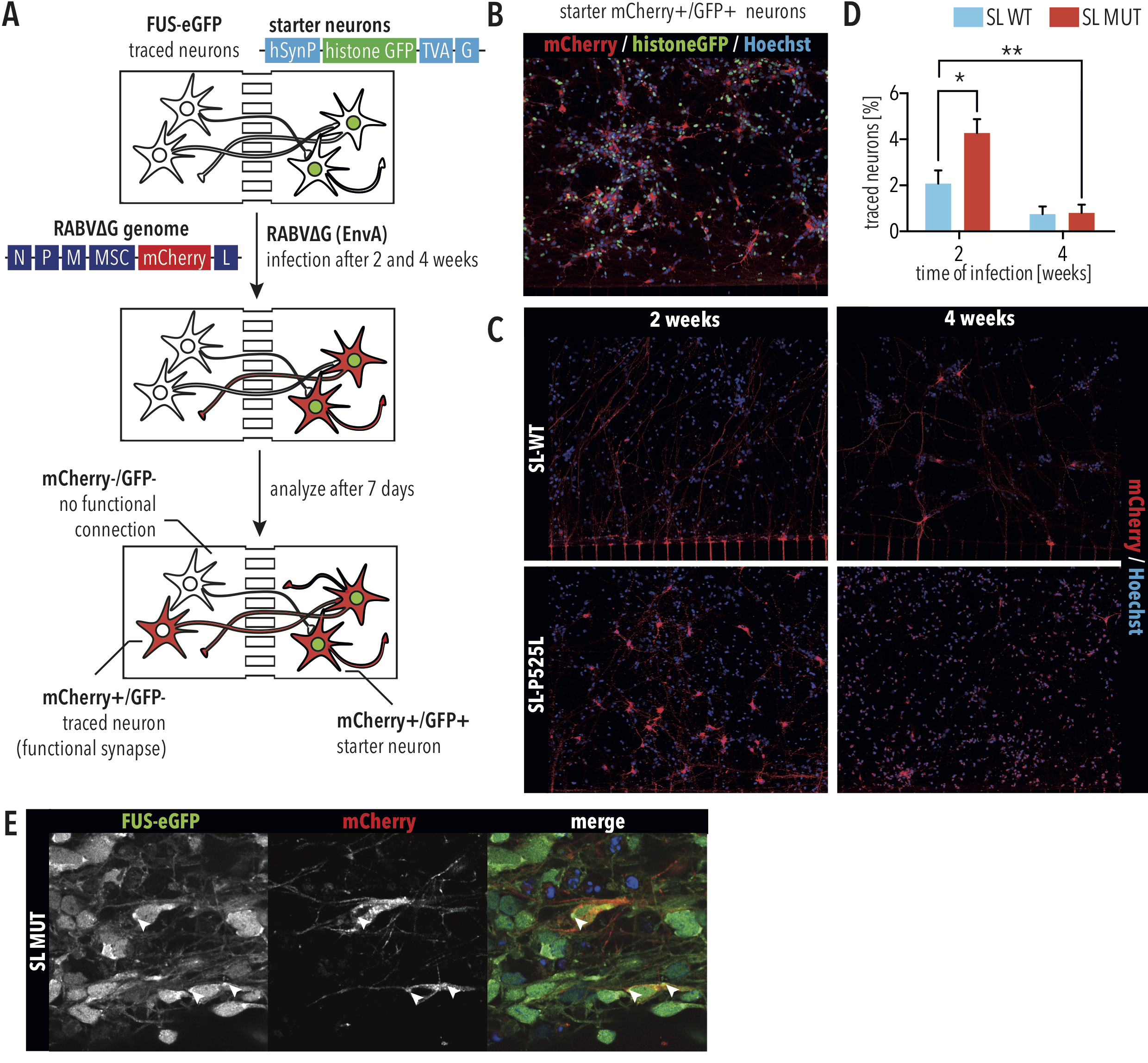

Supplement: FIGURE S2 — RABVΔG(EnvA) tracing exacerbated two pathological hallmarks of FUS-ALS. (A) Diagram illustrating monosynaptic tracing using RABVΔG(EnvA) and microfluidic chambers. (B) Starter neurons express the TVA receptor, H2B-GFP and rabies glycoprotein. Following infection with RABVΔG(EnvA), starter neurons also express mCherry. (C,D) P525L SL FUS-eGFP neurons show fewer traced cells 4 weeks compared with 2 weeks following infection as marked by RABVΔG(EnvA)-mCherry transgene expression. n = 2. Error bars indicate SEM. ∗ and ∗∗ correspond to p < 0.05 and 0.01, respectively, according to 2way ANOVA, Sidak post-test for multiple comparisons. (E) Traced neurons show FUS-eGFP granules. White arrows indicate neurons positive for stress granules and mCherry transgene expression. SL, short linker; MUT, mutant; WT, wild type; histone GFP, H2B-GFP. [file Image_2.TIFF]

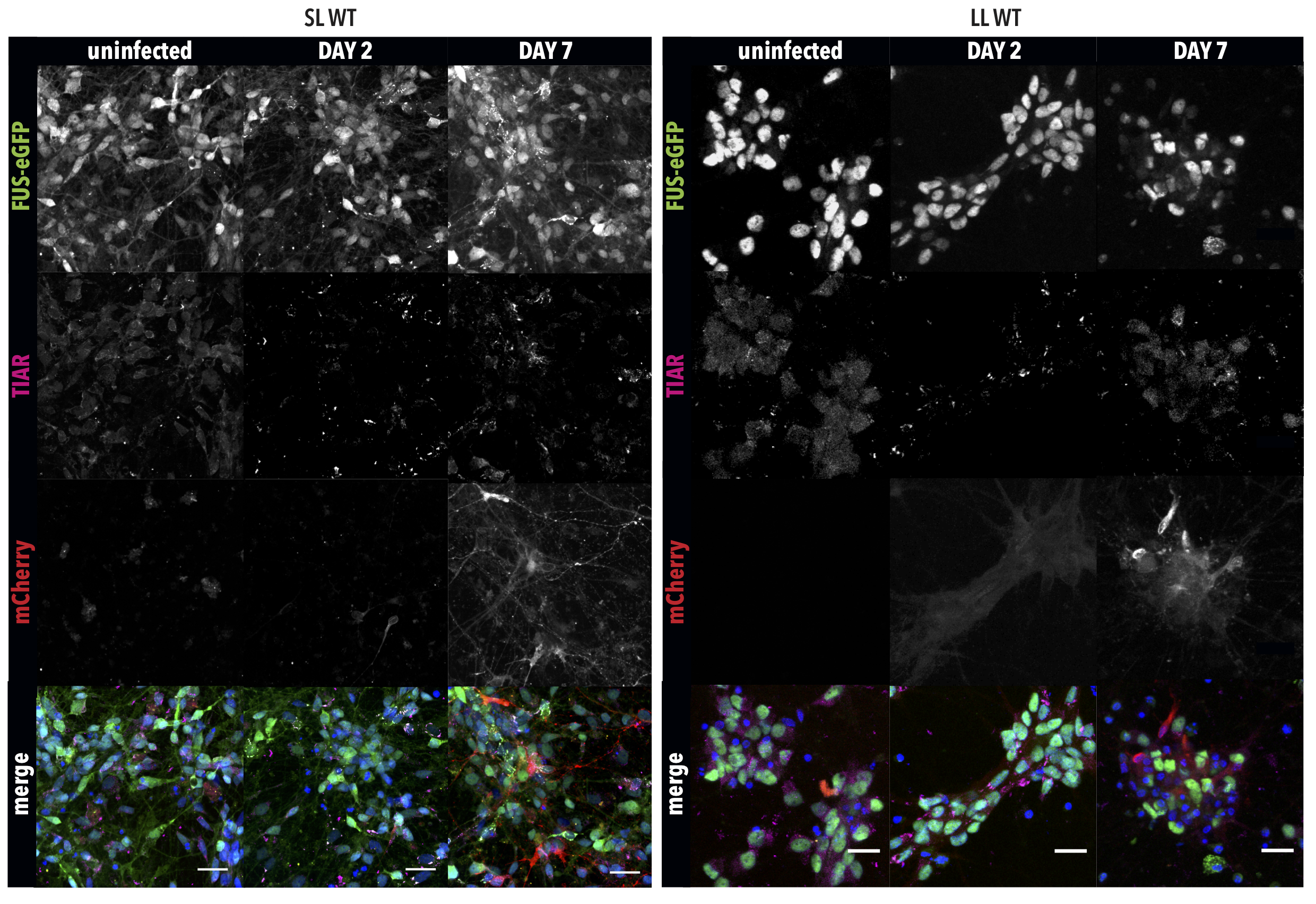

Supplement: FIGURE S3 — Direkt infection of LL and SL FUS-eGFP WT spinal neurons with RABVΔG-mCherry. 2 days following infection, FUS-WT spinal neurons show stress granules, and 7 days following infection, FUS-WT spinal neurons show RABVΔG-mCherry transgene expression. Scale bar = 25 μm. [file Image_3.TIFF]

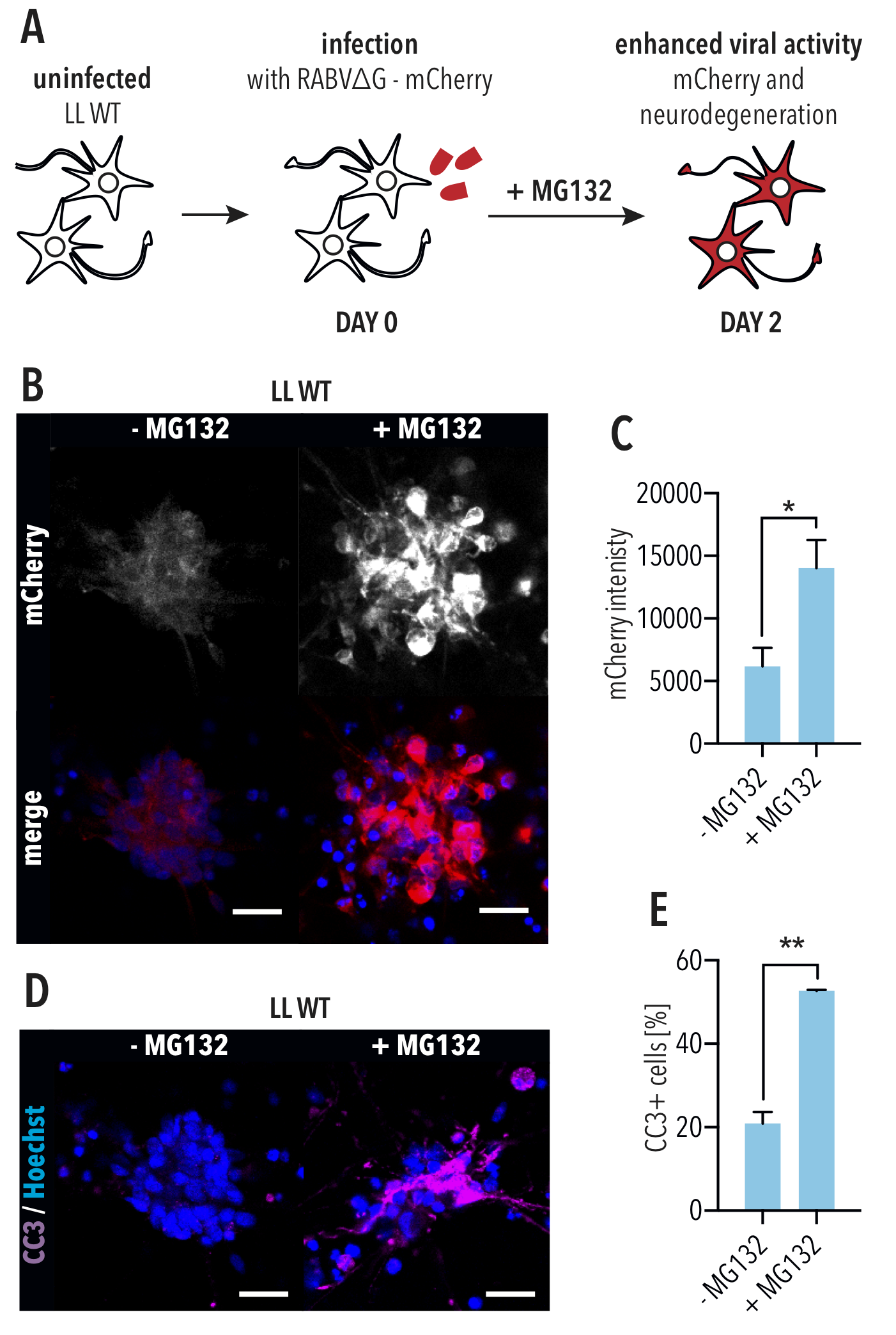

Supplement: FIGURE S4 — Proteasomal inhibition increases RABVΔG-mCherry levels and neurodegeneration. (A) Diagram illustrating infection of iPSC-derived spinal neurons with RABVΔG in the presence of 2.5 μM MG-132. (B,C) RABVΔG-mCherry levels are increased in iPSC-derived spinal neurons with LL WT FUS-eGFP at 2 days following infection of spinal neurons in presence of the proteasome inhibitor MG-132. (D,E) Cleaved-Caspase3 (CC3) levels are increased in iPSC-derived spinal neurons with LL WT FUS-eGFP at 2 days following infection of spinal neurons in presence of the proteasome inhibitor MG-132. Scale bar = 25 μm. n = 3. Error bars indicate SEM. ∗ and ∗∗ correspond to p < 0.05 and 0.01, respectively. LL, long linker; MUT, mutant; WT, wild type. [file Image_4.TIFF]

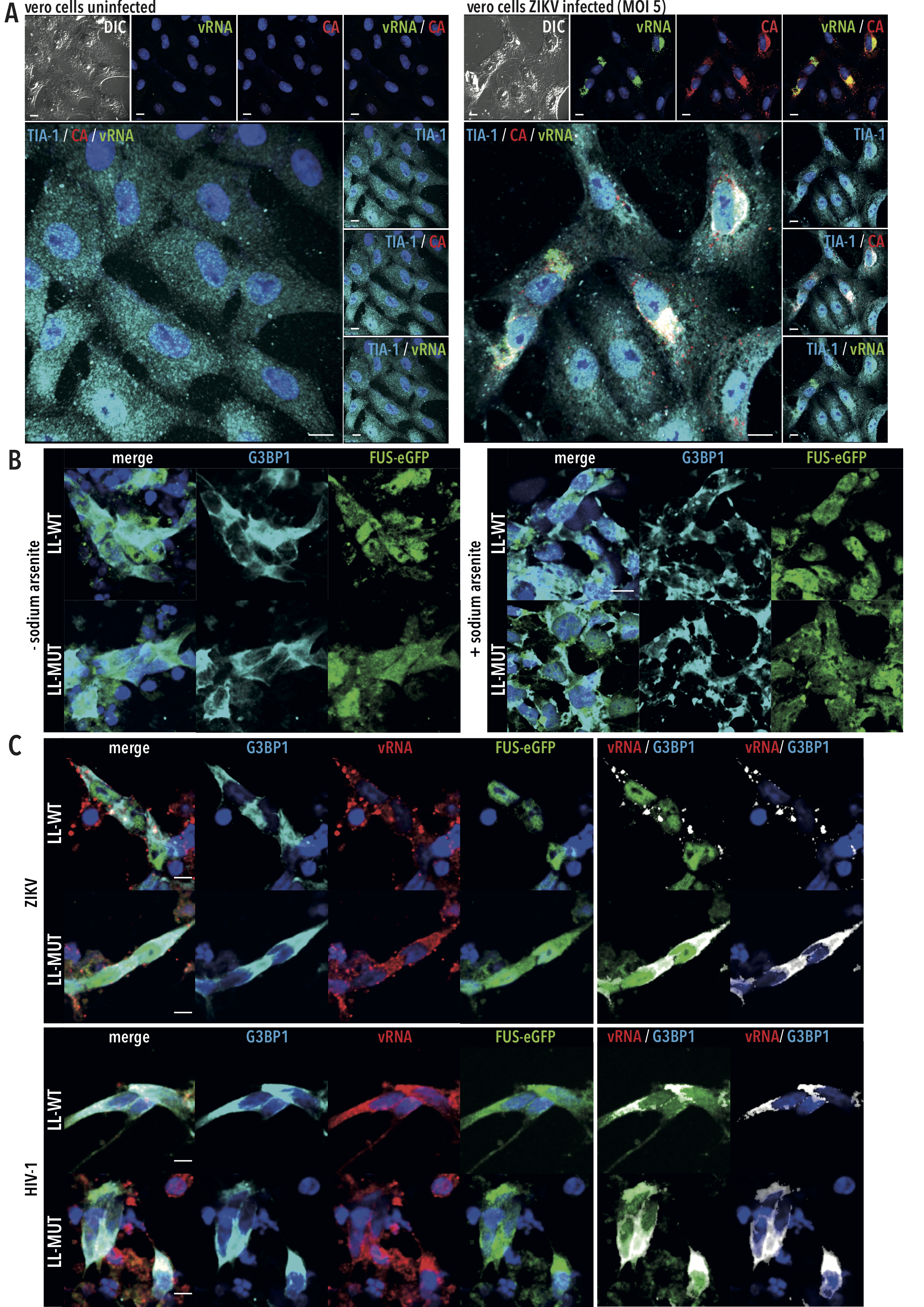

Supplement: FIGURE S5 — FUS-eGFP-positive SGs form following arsenite treatment but not HIV-1 or ZIKV infection. (A) J2 antibody was validated in Vero cells infected with the same viral stocks used in MNs. ZIKV infected Vero cells show colocalization of vRNA, capsid (CA) and TIAR at the nuclear periphery. Scale bar = 10 μm. (B) FUS-eGFP spinal neurons show FUS-eGFP and G3BP1 positive SGs 1 h after adding 500 μM arsenite. Scale bar = 5 μm. (C) Spinal neurons do not show FUS-eGFP granule formation after infection. The G3BP1 and vRNA in ZIKV infected WT-FUS cell colocalizes in cytoplasmic puctae. Co-localized G3BP1 and vRNA, are much more diffuse in the P525L FUS mutant infected by both ZIKV or HIV-1, as it is for HIV-1 infected FUS WT cells. Scale bar = 5 μm. MOI, multiplicity of infection; DIC, differential interference contrast; CA, ZIKV capsid; LL, long linker; MUT, mutant; WT, wild type; vRNA, viral RNA; /, colocalization. [file Image_5.TIFF]

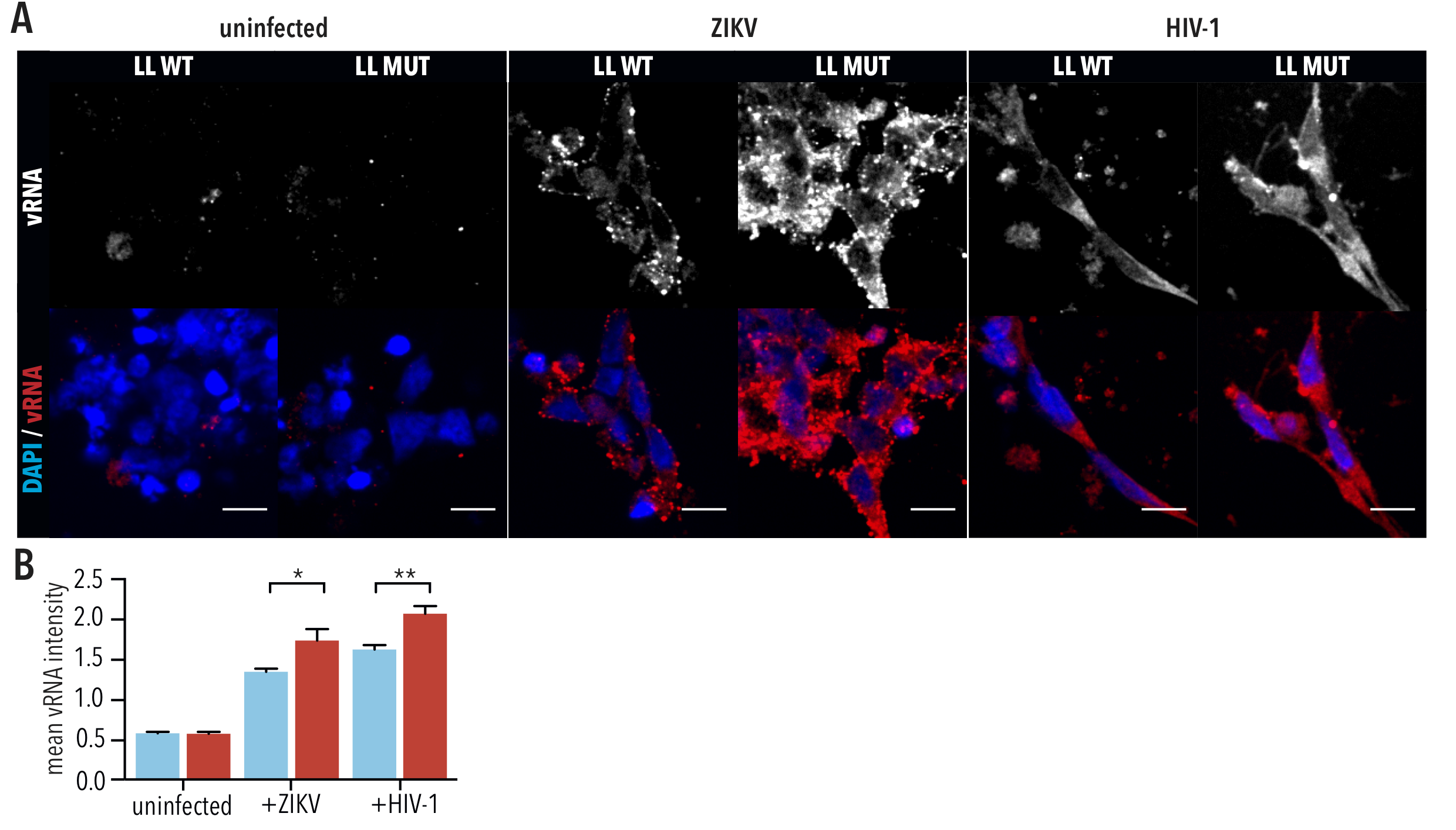

Supplement: FIGURE S6 — FUS-P525L spinal neurons show increased cytoplasmic vRNA levels (A,B) vRNA levels in FUS-P525L spinal neurons are increased in the cytoplasmic localization at the nuclear periphery after ZIKV or HIV-1 infection in comparison to WT. Scale bar = 10 μm. ∗ and ∗∗ indicate p < 0.01 and 0.0001, respectively, according to one-way ANOVA, Tukey post-test for multiple comparisons. n = 6. Error bars represent SEM. [file Image_6.TIFF]
